# Hierarchical NiCo_2_Se_4_ Arrays Composed of Atomically Thin Nanosheets: Simultaneous Improvements in Thermodynamics and Kinetics for Electrocatalytic Water Splitting in Neutral Media

**DOI:** 10.1002/advs.202402889

**Published:** 2024-06-18

**Authors:** Hongyu Chen, Yongsheng Xu, Xiaojie Li, Qing Ma, Delong Xie, Yi Mei, Guojing Wang, Yuanzhi Zhu

**Affiliations:** ^1^ Faculty of Chemical Engineering Yunnan Provincial Key Laboratory of Energy Saving in Phosphorus Chemical Engineering and New Phosphorus Materials Kunming University of Science and Technology Kunming Yunnan 650500 China; ^2^ School of Chemistry and Chemical Engineering/State Key Laboratory Incubation Base for Green Processing of Chemical Engineering Shihezi University Shihezi 832000 China; ^3^ PetroChina Shenzhen New Energy Research Institute Shenzhen 518052 China

**Keywords:** hierarchical ultrathin nanosheets, selenide, structural reconstruction, vacancies, water splitting

## Abstract

The inefficiency of electrocatalysts for water splitting in neutral media stems from a comprehensive impact of poor intrinsic activity, a limited number of active sites, and inadequate mass transport. Herein, hierarchical ultrathin NiCo_2_Se_4_ nanosheets are synthesized by the selenization of NiCo_2_O_4_ porous nanoneedles. Theoretical and experimental investigations reveal that the intrinsic hydrogen evolution reaction (HER) activity primarily originate from the NiCo_2_Se_4_, whereas the high oxygen evolution reaction (OER) performance is related to the NiCoOOH due to the structural reconstruction. The abundant Se and O vacancies introduced by atomically thin nanostructure modulate the electronic structure of NiCo_2_Se_4_ and NiCoOOH, thereby improving the intrinsic HER and OER activities, respectively. COMSOL simulation demonstrate the edges of extended nanosheets from the main body significantly promote the charge aggregation, boosting the reduction and oxidation current during HER/OER process. This charge aggregation effect notably exceeds the tip effect for the nanoneedle, highlighting the unique advantage of the hierarchical nanosheet structure. Benefiting from abundant vacancies and unique nanostructure, the hierarchical ultrathin nanosheet simultaneously improve the thermodynamics and kinetics of the electrocatalyst. The optimized samples display an overpotential of 92 mV for HER and 214 mV for OER at 100 mA cm^−2^, significantly surpassing the performance of currently reported HER/OER catalysts in neutral media.

## Graphical abstract

Hierarchical NiCo_2_Se_4_ arrays composed of atomically thin nanosheets grown on carbon fiber paper are first synthesized, which exhibit an outstanding hydrogen evolution reaction and oxygen evolution reaction performance and good durability in neutral media. Benefiting from abundant vacancies and unique nanostructure, the hierarchical ultrathin nanosheet simultaneously improve the thermodynamics and kinetics of the electrocatalyst.

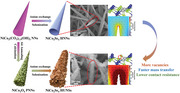

## 1 Introduction

Water electrolysis under neutral conditions, integrating hydrogen evolution reaction (HER) and oxygen evolution reaction (OER), presents a sustainable approach for clean hydrogen production, meeting the demand for unconstrained energy sources.^[^ ^1^, ^2^, ^3^ ^]^ This technology is versatile, including applications in neutral salts or buffers‐based water electrolysis,^[^ ^4^ ^]^ direct electrolytic seawater splitting,^[^ ^5^, ^6^ ^]^ photoelectrocatalysis,^[^ ^7^ ^]^ and bioelectrocatalysis,^[^ ^8^ ^]^ standing out for its environmental friendliness, minimal corrosion, cost‐effectiveness, and compatibility with catalysts sensitive to extreme conditions.^[^ ^9^, ^10^, ^11^ ^]^ However, current performance is limited by the slow kinetics and poor ionic conductivity in neutral electrolytes.^[^ ^12^, ^13^ ^]^ Unlike acidic or alkaline electrolytes, neutral conditions have fewer reactive H^+^ and OH^−^ ions, with these ions almost entirely originating from water dissociation. This necessitates a catalyst that can efficiently enrich and dissociate water molecule, as well as possess good mass transfer performance. These requirements highlight the critical need for simultaneous enhancements in both thermodynamics and kinetics of the catalysts for HER and OER.

Ultrathin non‐layered nanosheets distinct from conventional 2D nanosheets by the absence of layered structures in their bulk crystalline configuration.^[^ ^14^, ^15^, ^16^, ^17^ ^]^ This structure leads to significant structural distortion, leading to an abundance of dangling bonds and unsaturated surface atoms. These features enhance the catalytic sites exposure, optimize the chemisorption of reactants/intermediates and facilitate rapid interfacial charge transfer for electrocatalysis.^[^ ^18^, ^19^, ^20^ ^]^ However, most non‐layered nanosheets, synthesized through either top–down or bottom–up routes,^[^ ^17^, ^21^ ^]^ are in powder form and must be reprocessed into membrane electrodes assembly (MEA) using conductive agents and binders. This procedure frequently leads to the re‐stacking of 2D materials, consequently burying the active sites and impeding both electron and ion transport. Developing self‐standing 3D nanostructures composed of low‐dimensional sub‐units presents a viable solution. These arrays not only establish a binder‐free catalyst layer on gas diffusion layer with low contact resistance, but also create discontinuous three‐phase (solid–liquid–gas) contact lines to mitigate the bubble jamming effect, thereby enhancing water electrolysis performance particularly under high current densities.^[^ ^22^, ^23^, ^24^ ^]^ To data, various high‐performance nanoarray have been developed, including nanowire arrays, nanosheet arrays, biomimetic nanoarrays and heterostructure arrays. However, achieving array composed of atomically thin nanosheets and the intrinsic impact of this hierarchical structure on activity and mass transfer for electrocatalyst has not been thoroughly explored.

NiCo_2_Se_4_, emerging as non‐layered bimetallic selenides, has been extensively investigated as a water‐splitting catalyst, due to its high electronic conductivity, versatile redox nature, and hybrid d orbitals.^[^ ^25^, ^26^, ^27^, ^28^, ^29^ ^]^ For instance, Sancho et al. discovered that NiCo_2_Se_4_ nanowires grown on carbon fiber paper exhibit good OER performance in alkaline conditions.^[^ ^26^ ^]^ Janani et al. demonstrated the NiCo_2_Se_4_ nanoparticles as dual‐functional catalysis for both HER and OER in alkaline media.^[^ ^29^ ^]^ Despite these advancements, the catalytic performance of NiCo_2_Se_4_ is constrained by a limited number of active sites, suboptimal intrinsic activity, and slow mass transfer kinetics. Developing hierarchical ultrathin NiCo_2_Se_4_ nanosheets could address these limitations and substantially improve water splitting efficiency. Nonetheless, achieving such a structure is challenging due to the lack of an anisotropic growth driving force for the non‐layered structure.^[^ ^19^, ^20^ ^]^ Furthermore, comprehensive investigations into the activity origins and structural reconstruction of NiCo_2_Se_4_ under neutral conditions remain unexplored.

In this study, we report that the structure of NiCo_2_Se_4_ can be modulated through careful selection of precursor materials and control over the selenization process. The observed morphological variations of NiCo_2_Se_4_ arise from differences in the anion composition and porosity in the precursors, which potentially affect the Kirkendall effect during the process of anion exchange. Consequently, we successfully synthesized both hollow nanoneedles and hierarchical ultrathin nanosheets, enabling a comprehensive investigation of their origin of activity, thermodynamic and kinetic properties for electrocatalytic water splitting in neutral media. Theoretical and experimental investigations reveal the intrinsic HER activity originates from the NiCo_2_Se_4_ whereas the high OER performance is related to the NiCoOOH due to the structural reconstruction. Benefiting from abundant vacancies and unique nanostructure, the hierarchical ultrathin nanosheet simultaneously improve the thermodynamics and kinetics of the electrocatalyst. The optimized samples display an overpotential of 92 mV for HER and 214 mV for OER at current density of 100 mA cm^−2^, significantly surpassing the performance of currently reported HER/OER catalysts in neutral media.

## 2 Results and Discussion

### 2.1 Morphological and Structural Characterizations

**Figure 1:**
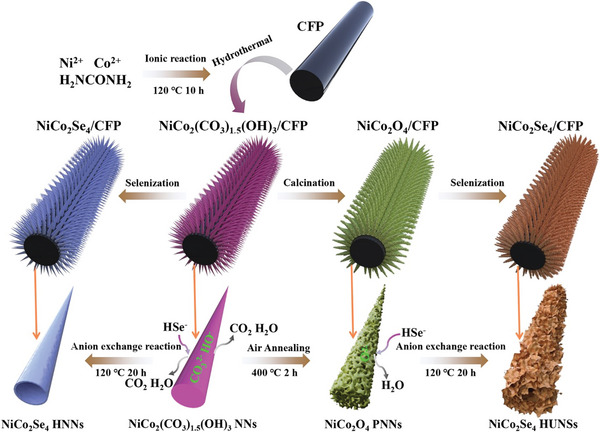
Schematic illustration of synthetic process of NiCo_2_Se_4_ HNNs/CFP and NiCo_2_Se_4_ HUNSs/CFP.

The fabrication of NiCo_2_Se_4_ HUNSs/CFP samples was achieved through a three‐step process as illustrated in **Figure** **1**. Carbon fiber paper (CFP) was chosen as the substrate for growth of nano‐catalyst due to its excellent electrical conductivity and 3D porous structure (SEM and XRD in Figure S1, Supporting Information). Initially, the NiCo_2_(CO_3_)_1.5_(OH)_3_ nanoneedles (NNs) were directly grown on the CFP through a hydrothermal reaction in a solution containing Ni(NO_3_)_2_·6H_2_O, Co(NO_3_)_2_·6H_2_O and urea. Subsequently, the porous NiCo_2_O_4_ nanoneedles (NiCo_2_O_4_ PNNs) was prepared via the calcination of NiCo_2_(CO_3_)_1.5_(OH)_3_ NNs in air atmosphere at 400 °C for 2 h. Finally, the hierarchical ultrathin NiCo_2_Se_4_ nanosheets with rich selenium vacancies (NiCo_2_Se_4_ HUNSs) were achieved through direct hydrothermal selenization of the NiCo_2_O_4_ PNNs using an anion exchange process. Meanwhile, hollow NiCo_2_Se_4_ nanoneedles (NiCo_2_Se_4_ HNNs) were also synthesized using NiCo_2_(CO_3_)_1.5_(OH)_3_ NNs as precursor by a similar hydrothermal selenization strategy.

**Figure 2:**
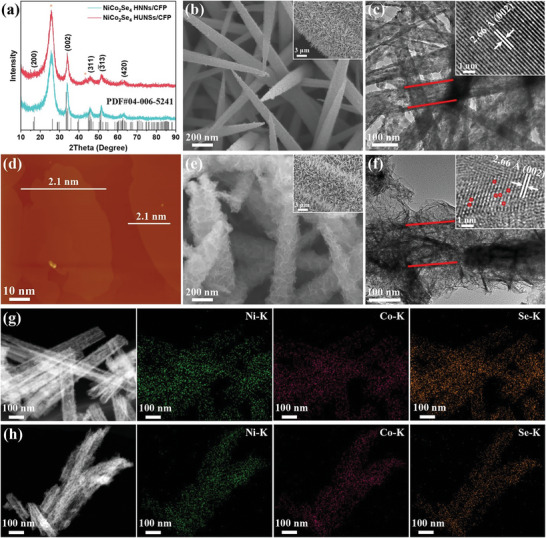
a) XRD spectra of NiCo_2_Se_4_ HNNs/CFP and NiCo_2_Se_4_ HUNSs/CFP. b,e) high‐magnification SEM, and c,f) TEM images of (b,c) NiCo_2_Se_4_ HNNs/CFP and (e,f) NiCo_2_Se_4_ HUNSs/CFP, respectively. d) The AFM image of NiCo_2_Se_4_ HUNSs; HAADF‐STEM and corresponding EDX elemental mapping images of g) NiCo_2_Se_4_ HNNs/CFP and h) NiCo_2_Se_4_ HUNSs/CFP. Insets: (b,e) the low‐magnification SEM and (c,f) HRTEM images of (b,c) NiCo_2_Se_4_ HNNs/CFP and (e,f) NiCo_2_Se_4_ HUNSs/CFP.

Scanning electron microscopy (SEM), transmission electron microscopy (TEM), and X‐ray diffraction (XRD) were used to characterize the morphologies, structures and composition. The SEM images, TEM image and XRD pattern in Figure S2 (Supporting Information) display the dense and uniform NiCo_2_(CO_3_)_1.5_(OH)_3_ nanoneedles with smooth surfaces, which are well‐aligned on the CFP. After calcination process, the cubic NiCo_2_O_4_ nanoneedles with highly porous features are uniformly grown on CFP (SEM, TEM, and XRD in Figure S3, Supporting Information). This highly porous structure results from the decomposition and volatilization of the unstable OH^−^ and CO_3_^2−^ groups in the NiCo_2_(CO_3_)_1.5_(OH)_3_ precursor during annealing. After undergoing the same hydrothermal selenization process, both the NiCo_2_(CO_3_)_1.5_(OH)_3_ NNs and NiCo_2_O_4_ PNNs were transformed into monoclinic NiCo_2_Se_4_ with identical X‐ray diffraction (XRD) patterns (**Figure** **2**a). Notably, the two kinds of NiCo_2_Se_4_ exhibit completely different morphologies. The NiCo_2_Se_4_ HNNs exhibit highly dense nanoneedle array evenly distributed over the CFP surface (Figure 2b). These nanoneedles are hollow, with a wall thickness of about 9–15 nm (Figure 2c; Figure S4a, Supporting Information), attributed to the Kirkendall effect during the selenization process. In contrast, the NiCo_2_Se_4_ HUNSs is needle‐like superstructure composed of ultrathin nanosheets (Figure 2e), these nanosheets are atomically thin and interwoven into a 3D hierarchical structure (Figure 2f; Figure S4b, Supporting Information). Atomic force microscopic (AFM) image further confirmed that the average thickness of these ultrathin nanosheets is about 2.1 nm for NiCo_2_Se_4_ HUNSs (Figure 2d).

To investigate the formation mechanism of NiCo_2_Se_4_ HUNSs, the XRD and SEM measurements of NiCo_2_O_4_ PNNs samples after selenization periods of 0.5, 1, 2, 6, 12, and 20 h were conducted. The XRD results (Figure S5, Supporting Information) demonstrate that NiCo_2_O_4_ PNNs gradually transforms into Ni─Co(OH)_2_ with a layered crystal structure in the alkaline NaHSe aqueous solution (pH approximately 9.6). Simultaneously, the Se anion exchange reaction occurs, leading to the formation of NiCo_2_Se_4_ ultra‐thin nanosheets. The SEM images (Figure S6, Supporting Information) confirmed a progressive development of ultra‐thin, layered nanosheets in the structure. The porous structure of NiCo_2_O_4_ may also promotes the reconstruction reaction. In contrast, the NiCo_2_(CO_3_)_1.5_(OH)_3_ precursor does not transform into Ni─Co(OH)_2_, resulting in a markedly different morphology between NiCo_2_Se_4_ HUNSs and NiCo_2_Se_4_ HNNs samples.

The HAADF‐STEM image and corresponding elemental mapping of NiCo_2_Se_4_ HNNs and NiCo_2_Se_4_ HUNSs demonstrate that Co, Ni, and Se are homogeneously distributed throughout the nanostructure (Figure 2g,h). Furthermore, STEM‐EDX (Figure S7, Supporting Information) and inductively coupled plasma‐atomic emission spectrometry (ICP‐AES) analyses consistently confirm that the average Ni/Co/Se atomic ratio of NiCo_2_Se_4_ HNNs is close to the stoichiometric ratio of 1:2:4, whereas this value for NiCo_2_Se_4_ HUNSs is 1:2:3.5. The high‐resolution TEM (HRTEM) image of NiCo_2_Se_4_ HNNs displays well‐resolved, perfectly ordered lattice fringes with an interplanar spacing of 0.266 nm (Figure 2c inset), corresponding to the (002) plane of NiCo_2_Se_4_. Conversely, the lattice fringes of NiCo_2_Se_4_ HUNSs are distinguishable in a few regions, indicating a defect‐rich nature of the nanosheets (Figure 2f inset). These observations suggest that selenization of the NiCo_2_O_4_ PNNs tends to create an amorphous microstructure and leads to the loss of some Se species in NiCo_2_Se_4_ HUNSs.

**Figure 3:**
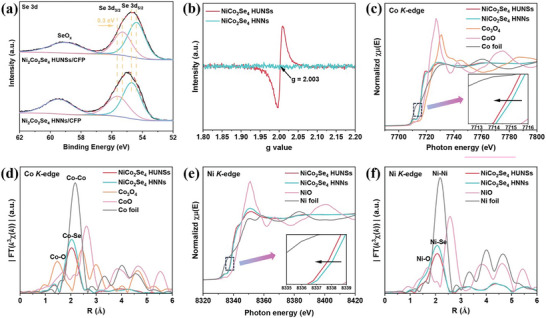
a) The high‐resolution Se 3d XPS spectra and b) EPR spectra of NiCo_2_Se_4_ HNNs/CFP and NiCo_2_Se_4_ HUNSs/CFP. c) Co K‐edge XANES spectra and d) the corresponding FT curves of the NiCo_2_Se_4_ HUNSs, NiCo_2_Se_4_ HNNs, standard Co_3_O_4_, standard CoO and Co foil. e) Ni K‐edge XANES spectra and f) the corresponding FT curves of the NiCo_2_Se_4_ HUNSs, NiCo_2_Se_4_ HNNs, standard NiO and Ni foil. the insets: (c,e) the enlarged view of the pre‐edge profiles.

The surface chemical state and composition of the NiCo_2_Se_4_ HNNs/CFP and NiCo_2_Se_4_ HUNSs/CFP were analyzed by X‐ray photoelectron spectroscopy (XPS) spectra. The XPS survey spectra for these two samples confirms the presence of Ni, Co and Se elements (Figure S8, Supporting Information). The high‐resolution Ni 2p, Co 2p, and Se 3d XPS spectra for the two samples are shown in **Figures** **3**a and S9 (Supporting Information). The high‐resolution Ni 2p (Figure S9a, Supporting Information) and Co 2p (Figure S9b, Supporting Information) spectra of NiCo_2_Se_4_ HUNSs exhibit a significant negative shift compared with NiCo_2_Se_4_ HNNs, implying the decrease in the oxidation state of Ni and Co due to the formation of Se vacancies. This is further confirmed by the higher atomic ratio of Ni^2+^/Ni^3+^ and Co^2+^/Co^3+^ in NiCo_2_Se_4_ HUNSs than in NiCo_2_Se_4_ HNNs, respectively.^[^ ^30^ ^]^ Furthermore, the Se 3d of NiCo_2_Se_4_ HUNSs exhibits three characteristic peaks at 54.38, 55.28 and 59.02 eV (Figure 3a), corresponding to the Se 3d_5/2_, Se 3d_3/2_, and SeO_x_, respectively.^[^ ^31^ ^]^ In concert with Ni and Co XPS, the binding energies of Se 3d_5/2_ and 3d_3/2_ peaks in NiCo_2_Se_4_ HUNSs shift to lower binding energy compared to those of NiCo_2_Se_4_ HNNs, further confirming the presence of Se vacancies.^[^ ^32^, ^33^ ^]^ Electron paramagnetic resonance (EPR) exhibits a signal peak at g = 2.003 (Figure 3b), originates from the unpaired electrons trapped by Se vacancies.^[^ ^34^, ^35^ ^]^ NiCo_2_Se_4_ HUNSs/CFP exhibits a much stronger symmetrical EPR signal compared to NiCo_2_Se_4_ HNNs/CFP, implying an abundance of Se vacancies in NiCo_2_Se_4_ HUNSs/CFP.

X‐ray absorption spectroscopy (XAS) was performed to investigate the electronic structure and local geometrical structures. The Co K‐edge X‐ray absorption near‐edge structure (XANES) spectra (Figure 3c) of NiCo_2_Se_4_ HNNs and NiCo_2_Se_4_ HUNSs are significantly different from that of standard Co_3_O_4_, CoO, and Co foil references, indicating that the chemical environment of Co in these samples are completely different from that of Co_3_O_4_, CoO, and Co foil. Furthermore, NiCo_2_Se_4_ HUNSs exhibit a negative shift in the rising‐edge region compared to that of NiCo_2_Se_4_ HNNs, which originated from the electron donation from electron‐rich Se vacancies, in line with the XPS results. Compared to those of NiCo_2_Se_4_ HNNs, the R space (Figure 3d) of the Co K edge‐extended X‐ray absorption fine structure (EXAFS) for NiCo_2_Se_4_ HUNSs exhibit noticeable decreases in peak intensity, which can be attributed to the missing Co−Se coordination. The optimal fitting results of the EXAFS spectra further demonstrate that the NiCo_2_Se_4_ HUNSs is coordination unsaturated (Figure S10a,b; Table S1, Supporting Information), with the Co–Se coordinative number of 4.8 for NiCo_2_Se_4_ HUNSs and 5.9 for NiCo_2_Se_4_ HNNs. The Ni XAS analysis for NiCo_2_Se_4_ HUNSs and NiCo_2_Se_4_ HNNs reflect the same trend (Figure 3e,f; Figure S10c,d and Table S1, Supporting Information). All results reveal that the use of NiCo_2_O_4_ PNNs as precursors for selenization can introduce rich V_Se_ into the products compared with the NiCo_2_(CO_3_)_1.5_(OH)_3_ NNs as precursor.

### 2.2 Electrocatalytic Performance

**Figure 4:**
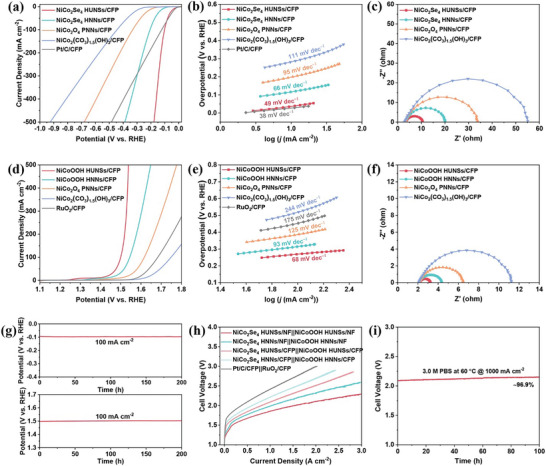
Electrocatalytic properties measurements in 1 m PBS. a) Polarization curves, b) Tafel plots, and c) Nyquist plots of the various catalyst samples for the HER. d) Polarization curves, e) Tafel plots, and f) Nyquist plots of the various catalyst samples for the OER. g) CP curves of (up) NiCo_2_Se_4_ HUNSs/CFP and (down) NiCoOOH HUNSs/CFP obtained at a constant j of (up) −100 mA cm^−2^ and (down) 100 mA cm^−2^. h) Current density‐cell voltage curve from the AEM electrolyzer at 60 °C in 3.0 m PBS. i) CP curves of the AEM electrolyzer at 60 °C under a 1000 mA cm^−2^ current density in 3.0 m PBS for 100 h.

The HER electrocatalytic performances of all the samples were evaluated in 1 m PBS solution. The polarization curves (**Figure** **4**a) show that NiCo_2_Se_4_ HUNSs/CFP electrode exhibits the highest activity with an over potential at 100 mA cm^−2^ (*η*_100_) of 102 mV, which is much lower than those of NiCo_2_Se_4_ HNNs/CFP (207 mV) and Pt/C/CFP (123 mV). The HER activity of the NiCo_2_Se_4_ HUNSs/CFP is superior to most of recently reported noble‐metal‐free HER electrocatalysts in neutral electrolyte (Table S2, Supporting Information). The Tafel slope of the NiCo_2_Se_4_ HUNSs/CFP is 49 mV dec^−1^ (Figure 4b), indicating a Volmer−Heyrovsky pathway where the Heyrovsky step is the rate‐determining step. Clearly, NiCo_2_Se_4_ HUNSs/CFP significantly accelerate the water dissociation process under neutral condition, thus paving a smoother pathway for subsequent reactions. In addition, the electrochemical impedance spectroscopy (EIS) measurement reveal that the charge transfer resistance (R_ct_) of NiCo_2_Se_4_ HUNSs/CFP (7.16 Ω) is significantly lower than that of NiCo_2_Se_4_ HNNs/CFP (16.87 Ω) (Figure 4c), suggesting superior interfacial charge‐transfer kinetics. The electrochemical double‐layer capacitances (C_dl_) of NiCo_2_Se_4_ HUNSs/CFP (87.73 mF cm^−2^) shows a considerable increase compared to NiCo_2_Se_4_ HNNs/CFP (41.01 mF cm^−2^) (Figure S11a−e, Supporting Information), indicating that the nanostructure of NiCo_2_Se_4_ HUNSs/CFP can provide more reaction interfaces and accessible active sites. The current density normalized by ECSA (*j*_ECSA_) further indicates that NiCo_2_Se_4_ HUNSs/CFP processes better intrinsic activity than NiCo_2_Se_4_ HNNs/CFP (Figure S11f, Supporting Information). These results indicate that the hierarchical ultrathin nanosheets nanostructure can simultaneously improve thermodynamics and kinetics for HER in neutral media.

To estimate the long‐term durability of NiCo_2_Se_4_ HUNSs/CFP, the chronopotentiometry and LSV tests at 1st and 5000th cycles were performed. No obvious decay occurs in the HER activity at constant current density of 100 mA cm^−2^ for 200 h (Figure 4g). The LSV polarization curve of NiCo_2_Se_4_ HUNSs/CFP after 5000 CV cycles nearly overlaps with the initial one (Figure S12, Supporting Information). Further investigations into the structural stability of NiCo_2_Se_4_ HUNSs/CFP after the long‐term HER stability test were conducted. The SEM images, TEM image, and XRD pattern (Figure S13, Supporting Information) confirm that the morphology and phase structure of NiCo_2_Se_4_ HUNSs/CFP remain unchanged after continuous operation for 50 h, demonstrating the superior HER durability of NiCo_2_Se_4_ HUNSs/CFP in neutral electrolyte. The XPS spectra (Figure S14, Supporting Information) indicate that the ratio of Ni^2+^/Ni^3+^ increased compared to that in pristine NiCo_2_Se_4_ HUNSs/CFP, while the chemical states of Co and Se remained almost unchanged. This may be because the Co and Se sites participate in the HER reaction, whereas the Ni sites are not directly involved and are partially reduced under the reduction potential during HER.

Transition metal chalcogenides are efficient OER pre‐catalysts that in situ transform into metal oxyhydroxides as real active materials under OER condition.^[^ ^36^, ^37^, ^38^ ^]^ In view of the NiCo_2_Se_4_ HUNSs/CFP after activation in alkaline solution exhibits better OER activity than pristine NiCo_2_Se_4_ HUNSs/CFP (Figure S15, Supporting Information), both NiCo_2_Se_4_ HNNs and NiCo_2_Se_4_ HUNSs underwent activation in 1 m KOH prior to OER testing. The OER performance of all the samples were measured in 1 m PBS electrolyte. Mirroring the HER results, NiCoOOH HUNSs/CFP displayed impressive OER activity, with the lowest *η*_100_ of 269 mV (Figure 4d) and Tafel slope of 68 mV dec^−1^ (Figure 4e), surpassing NiCoOOH HNNs/CFP (314 mV, 93 mV dec^−1^) and commercial RuO_2_/CFP (453 mV, 175 mV dec^−1^). This performance exceeds that of many recently reported noble‐metal‐free electrocatalysts in neutral electrolyte (Table S3, Supporting Information). Chronopotentiometric curve (Figure 4g) and LSV curve (Figure S16, Supporting Information) demonstrate the NiCoOOH HUNSs/CFP also has considerable long‐term durability. Additionally, the Rct of NiCoOOH HUNSs/CFP (1.10 Ω) is lower than that of NiCoOOH HNNs/CFP (2.22 Ω) (Figure 4f). ECSAs‐normalized polarization curves reveal that NiCoOOH HUNSs/CFP has higher intrinsic OER activity than NiCoOOH HNNs/CFP (Figure S17, Supporting Information). These findings emphasize the structural advantage of hierarchical ultrathin nanosheet in enhancing intrinsic activity and charge transport, a benefit proven in the HER process and equally effective for the OER process.

### 2.3 Flow‐Cell Water Electrolyzer Performance

To demonstrate the practical application potential of NiCo_2_Se_4_ HUNSs/CFP electrode for neutral water electrolysis, the flow‐cell electrolyzer using the NiCo_2_Se_4_ HUNSs/CFP as cathode and NiCoOOH HUNSs/CFP as anode was investigated under 3.0 m PBS with a flow rate of 30 mL min^−1^ at 60 °C (Figure S18, Supporting Information). Owing to its outstanding bi‐functionality, the polarization curves (Figure 4h) reveal that NiCo_2_Se_4_ HUNSs/CFP||NiCoOOH HUNSs/CFP flow‐cell required 2.09 V and 2.51 V to achieve current density of 1.0 A cm^−2^ and 2.0 A cm^−2^, respectively. Moreover, when CFP is replaced by NF as growth substrate, the HER and OER activities of the samples further improved (Figure S19, Supporting Information). The resulting samples display an overpotential of 92 mV for HER and 214 mV for OER at current density of 100 mA cm^−2^, markedly outperforming current reported HER/OER catalysts elsewhere (Tables S2 and S3, Supporting Information). The corresponding NiCo_2_Se_4_ HUNSs/NF||NiCoOOH HUNSs/NF‐based flow‐cell only requiring 1.85 V and 2.10 V to reach the current densities of 1.0 A cm^−2^ and 2.0 A cm^−2^, respectively, which is comparable to the best non‐precious metal based neutral flow‐cell. Furthermore, the flow‐cell maintains its performance over 100 h of continuous operation at a constant current density of 1 A cm^−2^ (Figure 4i). These results demonstrate the suitability of NiCo_2_Se_4_ HUNSs for practical water splitting applications in neutral media.

### 2.4 Identifying OER Active Phase

**Figure 5:**
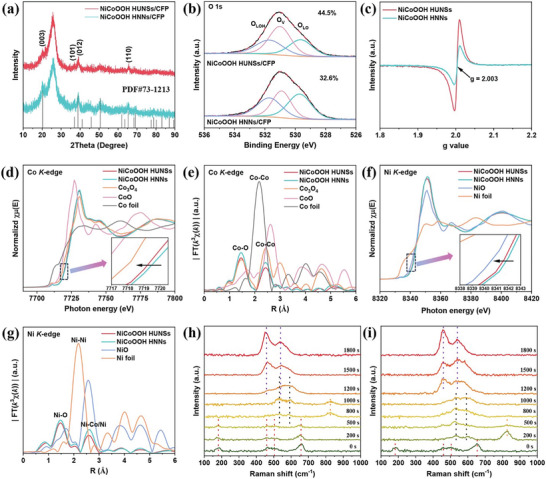
a) The XRD, b) high‐resolution O 1s XPS, and c) EPR spectra of NiCoOOH HNNs/CFP and NiCoOOH HUNSs/CFP. d) Co K‐edge XANES spectra and e) the corresponding FT curves of the NiCoOOH HUNSs, NiCoOOH HNNs, standard Co_3_O_4_, standard CoO and Co foil. f) Ni K‐edge XANES spectra and g) the corresponding FT curves of the NiCoOOH HUNSs, NiCoOOH HNNs, standard NiO and Ni foil. the insets: (d,f) the enlarged view of the pre‐edge profiles. In situ Raman spectra of h) NiCo_2_Se_4_ HNNs and i) NiCo_2_Se_4_ HUNSs at different chronopotentiometry test time from 0 to 1800 s at a constant j of 10 mA cm^−2^ for alkaline activation in 1 m KOH.

As NiCo_2_Se_4_ underwent reconstruction after alkaline activation or during the OER process, we further investigated the activity origin of NiCo_2_Se_4_ for OER. The XRD result reveal that both NiCo_2_Se_4_ HNNs and NiCo_2_Se_4_ HUNSs were transformed into crystalline NiCoOOH, while still maintaining their original morphology (**Figure** **5**a; Figure S20, Supporting Information). EDS measurements (Figure S21, Supporting Information) reveal that the initial Se component in NiCo_2_Se_4_ was almost entirely lost. Different from NiCo_2_Se_4_, which exhibited mixed valence states of +2 and +3 for both Co and Ni, the NiCoOOH samples exhibit predominantly Ni^3+^ and Co^3+^ oxidation states, as evidenced by Ni 2p XPS (Figure S22a, Supporting Information) and Co 2p XPS (Figure S22b, Supporting Information). These results confirm the complete reconstruction from NiCo_2_Se_4_ to NiCoOOH. The binding energies of Ni 2p and Co 2p for NiCoOOH HUNSs negatively shifts compared to those for NiCoOOH HNNs, suggesting an increase in oxygen vacancies. The O 1s of NiCoOOH HUNSs and NiCoOOH HNNs exhibit three characteristic peaks at 529.7, 530.9, and 531.7 eV (Figure 5b), corresponding to the lattice O (O_LO_), oxygen vacancy (O_v_) and lattice OH (O_LOH_), respectively. The concentration of O_v_ in NiCoOOH HUNSs (44.5%) is much higher than in NiCoOOH HNNs (32.6%). Moreover, compared with NiCoOOH HNNs, a stronger symmetrical EPR signal (g = 2.003), which originates from the unpaired electrons at oxygen vacancy sites, is observed for NiCoOOH HUNSs (Figure 5c).

The electronic and local coordination structures were further investigated by XAFS. As shown in the Co K‐edge XANES (Figure 5d), the absorption edge of NiCoOOH HNNs and NiCoOOH HUNSs shifts toward higher energy relative to that of Co foil, CoO and Co_3_O_4_, suggesting a Co valence state closed to +3. The rising‐edge region at the Co K edge of NiCoOOH HUNSs has a negative shift compared to that of NiCoOOH HNNs, attributed to the electron donation from oxygen vacancies. In addition, the main peak of Co K‐edge FT‐EXAFS spectra (Figure 5e) for NiCoOOH HUNSs displays obvious decreases relative to those of NiCoOOH HNNs, ascribing to the reduced O coordination number of Co sites due to the existence of rich O vacancies. The fitting results of the R space further confirm that the CoO_6_ octahedrons of NiCoOOH HUNSs are coordinative unsaturated (Figure S23a,b; Table S4, Supporting Information). The coordinative numbers of Co─O (4.6) and Co─M (M = Co, Ni) (3.1) are much lower than theoretical values of 6, suggesting the CoO_6_ octahedrons in NiCoOOH HUNSs are coordinative unsaturated. These values are also lower than the those for NiCoOOH HNNs (5.1 and 3.9, respectively). The Ni XAS analysis for both NiCoOOH HUNSs and NiCoOOH HNNs reflect similar trend (Figure 5f,g; Figure S23c,d and Table S4, Supporting Information). These results indicate that the hierarchical ultrathin nanosheets promote the formation of abundant edges and O vacancies during the reconstruction process.

To gain a deeper understanding of the reconstruction process, we conducted in situ Raman investigation on the evolution of NiCo_2_Se_4_ HNNs and NiCo_2_Se_4_ HUNSs under alkaline OER activation. Initially, the NiCo_2_Se_4_ HNNs sample displayed four characteristic Raman peaks at F_2g_ (183 cm^−1^), E_2g_ (458 cm^−1^), F_2g_ (502 cm^−1^), and A_1g_ (652 cm^−1^), corresponding to the phonon modes of NiCo_2_Se_4_ (Figure 5h).^[^ ^39^ ^]^ After CP test for 500s, these peaks gradually disappeared, indicating the breakdown of M─Se bonds and leaching of Se. From 800 to 1200 s, new peaks at 532 and 590 cm^−1^ appeared, suggesting the formation of NiCo(OH)_2_ species.^[^ ^40^ ^]^ Additionally, a new peak at 828 cm^−1^, assignable to selenate (SeO_4_^2−^),^[^ ^38^ ^]^ appeared and then faded from 800 to 1200 s, implying the substitution reaction between O and Se is completed within this interval. From 1200 to 1800 s, the peaks of metal hydroxide diminished and that of NiCoOOH increased, indicating the transformation of metal hydroxide into metal oxyhydroxides. Remarkably, this conversion from metal selenides to metal oxyhydroxides took 1500 s. A similar but faster reconstruction process was observed with NiCo_2_Se_4_ HUNSs (Figure 5i). The breakdown of M─Se bonds and Se leaching occurred within 200 s, followed by a conversion to metal hydroxide and ultimately to metal oxyhydroxide, completing within 1200 s. Notably, due to their unique hierarchical ultrathin nanosheet architecture, NiCo_2_Se_4_ HUNSs underwent reconstruction more readily than NiCo_2_Se_4_ HNNs, with a shorter conversion time to NiCoOOH. These Raman results clearly demonstrate the dynamic reconstruction and active species evolution for NiCo_2_Se_4_.

### 2.5 HER and OER Enhancement Mechanism

**Figure 6:**
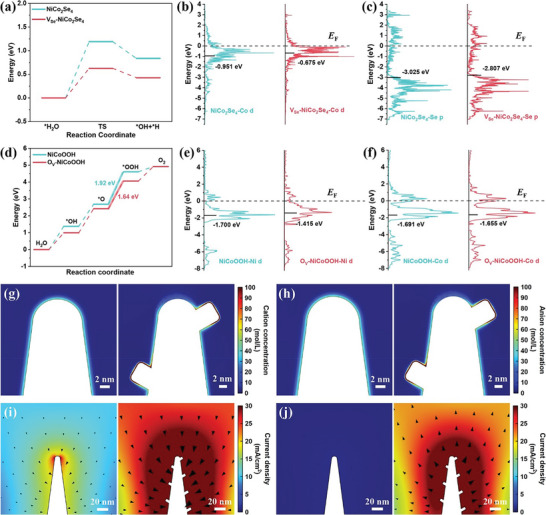
a) Reaction energy diagram of water dissociation into H^*^ and OH^*^ on the NiCo_2_Se_4_ (001) and V_Se_‐NiCo_2_Se_4_ (001) surfaces. The Partial density of states (PDOS) of the b) Co d‐orbital and c) Se p‐orbital at the adsorption sites on NiCo_2_Se_4_ (001) and V_Se_‐NiCo_2_Se_4_ (001). d) The free energy diagram of OER on the NiCoOOH (001) and O_v_‐NiCoOOH (001). The Partial density of states (PDOS) of the e) Ni d‐orbital and f) Co d‐orbital at the adsorption sites on NiCoOOH (001) and O_v_‐NiCoOOH (001). g) Cation concentration distribution and h) anion concentration distribution on the surface of nanoneedle (left) and hierarchical ultrathin nanosheet (right). i) Reduction current density distributions and j) oxidation current density distributions on the surface of nanoneedle (left) and hierarchical ultrathin nanosheet (right).

To explore the intrinsic impact of Se and O vacancies on the HER and OER catalytic activities of NiCo_2_Se_4_ and NiCoOOH, respectively, density functional theory (DFT) calculations were performed. The intact NiCo_2_Se_4_ (001) and V_Se_‐NiCo_2_Se_4_ (001) with selenium vacancy were modeled based on the XRD and HRTEM data (Figure S24, Supporting Information). The optimized HER process are exhibited in Figure S25 (Supporting Information). The H_2_O molecule tend to be adsorbed on Co atoms rather than Ni atoms, indicating a significant role of Co atoms in water activation. The water adsorption energy on Co atoms in V_Se_‐NiCo_2_Se_4_ (−0.56 eV) is significantly lower than NiCo_2_Se_4_ (−0.30 eV) (Figure S26, Supporting Information). The V_Se_‐NiCo_2_Se_4_ (0.63 eV) also exhibits lower energy barrier for water dissociation compared to NiCo_2_Se_4_ (1.19 eV) (**Figure** **6**a). These results suggest that the introduction of Se vacancy facilitate the water activation and dissociation. After water dissociation, the formed OH^*^ and H^*^ tends to be adsorbed on the Co atom and Se atom, respectively, following by a Heyrovsky step to form H_2_. We further calculated free energy of hydrogen adsorption (ΔG_H*_) on NiCo_2_Se_4_ and V_Se_‐NiCo_2_Se_4_ surfaces (Figure S27, Supporting Information). The ΔG_H*_ of V_Se_‐NiCo_2_Se_4_ (0.19 eV) is closer to thermoneutral value (zero) compared with NiCo_2_Se_4_ (ΔG_H*_ = 0.39 eV), implying the adsorption of H is optimized by introduction of Se vacancy. Charge density distribution, Bader charge, and densities of states (DOS) analysis further elucidate how Se vacancy modulate the electronic structure (Figure 6b,c; Figures S28–S30 and Table S5, Supporting Information). After the introduction of Se vacancy, Bader charge values increase from −0.126 to −0.081 for the Ni1 atom and from −0.163 to −0.132 for the Co1 atom. Meanwhile, the Bader charge for the Se1 atom decrease from 0.215 to 0.167. These results indicate a charge transfer from the Se vacancy to adjacent Ni, Co, and Se atoms in NiCo_2_Se_4_ HUNSs (Figures S28 and S29 and Table S5, Supporting Information), consistent with the XAS results. The V_Se_‐NiCo_2_Se_4_ also has stronger DOS near the Fermi level, suggesting V_Se_‐NiCo_2_Se_4_ has higher carrier concentration for improved electrical conductivity (Figure S30, Supporting Information). According to d‐ and p‐band theories, upward shifts in these bands lead to stronger binding interaction between the catalysts and adsorbates. As evidenced by partial density of states (PDOS) (Figure 6b,c), the d‐band center of Co atom and p‐band center of Se atom on V_Se_‐NiCo_2_Se_4_ exhibit a significant lift toward the Fermi level, which explain the improved H_2_O activation and ΔG_H*_ induced by selenium vacancy.

For OER process, the stability of NiCoOOH (001) surfaces with the different Ni atoms doping positions and O_v_‐NiCoOOH (001) with different O vacancy positions were assessed according to their surface energies from the DFT calculations (Figure S31, Supporting Information). The models of the most stable surface of NiCoOOH (001) and O_v_‐NiCoOOH (001) were selected for further analysis (Figure S32, Supporting Information). The optimized OER process are presented in Figure S33 (Supporting Information). According to the calculation, the Ni and Co sites are identified as the active sites for adsorbing OER intermediates (^*^OH, ^*^O, and ^*^OOH). As shown in Figure 6d, the free energies of OER intermediates on O_v_‐NiCoOOH are relatively lower than on NiCoOOH, and the O–O coupling process is the rate‐determining step (RDS). Specifically, the energy barrier of the RDS is reduced from 1.92 to 1.64 eV after introduction of oxygen vacancy. Similar to the effect of selenium vacancy, oxygen vacancy also leads to the increase of the charge density of surrounding Ni/Co/Se atoms, as confirmed by charge density distribution and Bader charge analysis in Figures S28 and S29 and Table S5 (Supporting Information). The DOS analysis in Figure S34 (Supporting Information) reveals that some new electronic states appear near the Fermi level after the introduction of oxygen vacancies, leading to higher electrical conductivity of O_v_‐NiCoOOH. Figure 6e,f reveals that the d‐band center of Ni (−1.415 eV) and Co (−1.655 eV) in O_v_‐NiCoOOH are closer to the Fermi level compared to NiCoOOH (−1.700 eV for Ni and −1.691 eV for Co), suggesting an elevated antibonding energy state for O_v_‐NiCoOOH. The higher antibonding energy state would lead to stronger binding interaction between the catalysts and adsorbates (Figure S35, Supporting Information).^[^ ^41^ ^]^ The d‐band center results above are consistent with the free energy diagram of OER, demonstrating the O_v_‐NiCoOOH has a stronger adsorption for the OER intermediates, thus reducing the energy barrier of the RDS. Overall, these results clearly confirm that the introduction of O vacancy can significantly modulate the electronic structure of the catalyst, optimize the energy of each OER step, and finally improve the activity of OER.

COMSOL multiphysics simulations were employed to investigate the influence of morphological structure on mass transfer in electrocatalytic HER and OER processes. The bare nanoneedle and nanoneedle with extended nanosheet were designed by finite element modeling, with their dimensions aligned to those observed in the TEM images. According to Figure S36 (Supporting Information), hierarchical nanosheet demonstrates a significantly stronger electric field intensity than nanoneedle, which may promote the accumulation of cation/anion on the surface of hierarchical nanosheet. Unlike in acidic or alkaline environments, the neutral electrolyte (1 m PBS solution) lacks free H^+^/OH^−^ ions. During HER or OER process, the migration of K^+^ or H_2_PO_4_^−^ ions in electric field lead to the aggregation of H_2_O on the catalyst surface, promoting the dissociation of H_2_O. Therefore, we simulated the ion environment under neutral condition by employing 1 m ions with +1 and −1 valence state to represent K^+^/H^+^ and H_2_PO_4_^−^/OH^−^ ions, respectively. The influence of the electric field on surface‐adsorbed cation/anion concentration was investigated by mapping the charge density in the Helmholtz layer adjacent to the electrode surface. Figure 6g,h shows a significant increase in charge density at the hierarchical nanosheets, especially along the edges of extended nanosheets from the main pole. The aggregation of these ions results in an increase in reduction and oxidation current. As shown in Figure 6i,j, the current density primarily accumulates at the top of the nanoneedle due to the tip effect. In contrast, the current density of hierarchical nanosheets significantly increases at the same applied voltage. These results suggest that the unique hierarchical nanosheet structure induce the redistribution of electric field environment, which promote ion enrichment on the edge of the extended nanosheets (Figure S37, Supporting Information), thus facilitating the electrocatalysis.

To validate the conclusions from the simulations, we performed a 120‐s water electrolysis using NiCo_2_Se_4_ HNNs and NiCo_2_Se_4_ HUNSs at −0.1 V for HER, and NiCoOOH HNNs and NiCoOOH HUNSs at 1.5 V for OER,^[^ ^42^, ^43^, ^44^ ^]^ as depicted in Figure S38a (Supporting Information). After electrolysis, with the potential still applied, all electrodes were removed from the cell. The potential was then discontinued, and the working electrode was rinsed by immersion in 10 mL of ultrapure water. The concentration of K^+^ or H_2_PO_4_^−^ ions on the electrode surface was subsequently measured using ICP analysis. As shown in Figure S38b,c (Supporting Information), the concentrations of K^+^ (for HER) and H_2_PO_4_^−^ (for OER) on the NiCo_2_Se_4_ HUNSs and NiCoOOH HUNSs electrodes were 1.92 times and 2.08 times higher, respectively, than those on the NiCo_2_Se_4_ HNNs and NiCoOOH HNNs electrodes, consistent with the COMSOL result.

## 3 Conclusion

In summary, we developed a hierarchical NiCo_2_Se_4_ nanostructure composed of atomically thin nanosheets by employing porous NiCo_2_O_4_ as precursor during the selenization process. Compard to conventional NiCo_2_Se_4_ hollow nanoneedles, our hierarchical nanosheets markedly improve both the thermodynamic and kinetic properties for electrocatalytic water splitting in neutral media. Theoretical and experimental investigations reveal that the intrinsic HER activity primarily attributed to the Co atoms and the presence of abundant Se vacancies in NiCo_2_Se_4_, while the exceptional OER performance is related to the Ni and Co atoms in NiCoOOH with abundant O vacancies due to the structural reconstruction. COMSOL simulation indicate that the edges of extended nanosheets from the main body significantly promote the charge aggregation, boosting the reduction and oxidation current during HER/OER process. This charge aggregation effect significantly surpasses the tip effect, highlighting the unique advantage of the hierarchical nanosheet structure. As a result, the optimized samples display excellent HER/OER activity and flow‐cell performance surpassing currently reported HER/OER catalysts in neutral media. This work highlights the crucial role of structure engineering of free‐standing nanoarrays for enhancing electrocatalytic activity in neutral media.

Electronic supplementary information (ESI) available: More experimental details and additional characterization results including SEM, TEM, XRD, HRTEM, XPS, DFT calculations and electrochemical data.

## 4 Experimental Section

### Synthesis of NiCo_2_(CO_3_)_1.5_(OH)_3_ NNs/CFP

Typically, NiCo_2_(CO_3_)_1.5_(OH)_3_ NNs/CFP was fabricated by using a simple hydrothermal reaction. The carbon fiber paper (CFP, 1 × 3 cm^2^) was thoroughly washed with acetone and deionized water by sonication sequentially for 15 min and then pretreated with concentrated nitric acid at the 75 °C for 90 min to achieve the surface hydroxylation of CFP. After being washed with water, the pretreated CFP was used as the support for the synthesis of integrated catalyst. First, Co(NO_3_)_2_·6H_2_O (0.349 g, 1.2 mmol), Ni(NO_3_)_2_·6H_2_O (0.174 g, 0.6 mmol), and urea (0.360 g, 6 mmol) were dissolved in 40 mL deionized water under vigorous stirring for 30 min to prepare the homogeneous solution. Subsequently, a piece of pretreated CFP and the solution were loaded into a 50 mL Teflon‐lined stainless steel autoclave. The autoclave was sealed and heated at 120 °C for 10 h and subsequently cooled down to room temperature naturally. Then, the as‐prepared NiCo_2_(CO_3_)_1.5_(OH)_3_ NNs/CFP was taken out and washed three times alternatively with water and ethanol, followed by drying at 60 °C for 12 h in vacuum oven.

### Synthesis of NiCo_2_O_4_ PNNs/CFP

To synthesize NiCo_2_O_4_ PNNs/CFP, the purified NiCo_2_(CO_3_)_1.5_(OH)_3_ NNs/CFP was heated to 400 °C at a ramping rate of 5 °C min^−1^ and then kept at this temperature for 2 h under air in a horizontal tube furnace. After naturally cooling to room temperature, the obtained NiCo_2_O_4_ PNNs/CFP were washed three times with deionized water and ethanol, followed by drying at 60 °C for 12 h under vacuum.

### Synthesis of NiCo_2_Se_4_ HNNs/CFP

A NiCo_2_Se_4_ HNNs/CFP electrode was synthesized by a hydrothermal selenization process. Initially, NaBH_4_ (40 mg, 1.06 mmol) and selenium powder (39 mg, ∼0.49 mmol) were dissolved in 1 mL of Ar‐saturated water in a glass vial and allowed to react for 30 min at 0 °C, yielding a clear NaHSe solution. Subsequently, a piece of as‐prepared NiCo_2_(CO_3_)_1.5_(OH)_3_ NNs/CFP was placed in a 50 mL Teflon‐lined stainless steel autoclave containing 30 mL water. The solution within the autoclave was purged with Ar gas for 1 h, after which the freshly prepared NaHSe solution was introduced into the autoclave. The autoclave was then sealed and maintained at 120 °C for 20 h. The resultant NiCo_2_Se_4_ HNNs/CFP was removed, purified by washing with deionized water and ethanol three times, and dried at 60 °C under vacuum for 12 h.

### Synthesis of NiCo_2_Se_4_ HUNSs/CFP

To fabricate NiCo_2_Se_4_ HUNSs/CFP, the as‐prepared NiCo_2_O_4_ PNNs/CFP underwent the same hydrothermal selenization process as described above. The resulting NiCo_2_Se_4_ HUNSs/CFP was removed, washed with deionized water and ethanol three times, and dried at 60 °C for 12 h under vacuum.

### Electrochemical Measurements

All electrochemical measurements were tested in a three‐electrode electrochemical workstation (CHI660D, CH Instruments, Inc.) in H_2_ or O_2_‐saturated 1.0 m PBS solution. The as‐prepared catalyst/CFP (1 cm^2^ geometric area), Ag/AgCl (saturated KCl solution) and graphite rod (for HER) or Pt wire (for OER) were used as the working electrode, reference electrode and counter electrode, respectively. For comparison, the commercial Pt/C or RuO_2_ with same mass loading was loaded onto CFP by drop casting a catalyst ink to prepare the control electrodes. All measured potentials in this work were referenced to RHE according to the Nernst equation: V_RHE_ = V_Ag/AgCl_ + E_Ag/AgCl_ + 0.059 pH. Linear scan voltammetry (LSV) curves were recorded at a sweep rate of 5 mV s^−1^. Electrochemical impedance spectroscopy (EIS) was obtained by sampling 100 points in the frequency range of 100 kHz–0.01 Hz with an AC voltage of 5 mV at a given potential. A chronopotentiometry experiment at a constant current density of 100 mA cm^−2^, and LSV before and after 5000 CV cycles were performed for durability testing. The electrochemically active surface areas (ECSAs) of various catalysts were evaluated using the measured electrochemical double layer capacitance (C_dl_) that is determined by CV measurements within the potential window of 0.2–0.3 V or 0.9–1.0 V, and sweep rates at 10, 20, 40, 60, 80 and 100 mV s^−1^ were chosen. All polarization curves were iR‐corrected to compensate for the effect of electrolyte resistance.

The two‐electrode anion exchange membrane (AEM, Sustainion X37‐FA) electrolyzer was assembled using NiCo_2_Se_4_ HUNSs/CFP or NiCo_2_Se_4_ HUNSs/NF (1.0 × 1.0 cm^2^) and NiCoOOH HUNSs/CFP or NiCoOOH HUNSs/NF (1.0 × 1.0 cm^2^) as cathode and anode, respectively. The Pt/C and RuO_2_ catalysts was also sprayed on the CFP as control electrodes by airbrush. The experiments were performed in 3.0 m PBS electrolytes under a constant flow rate of 30 mL min^−1^ at 60 °C.

### Computational Methods

All calculations were carried out using first principle density functional theory (DFT) by Vienna ab initio simulation package (VASP).^[^ ^45^ ^]^ The projector‐augmented wave (PAW) method was used to describe the interaction between ionic cores and valence electrons.^[^ ^46^ ^]^ The generalized gradient approximation (GGA) with the Perdew–Burke–Ernzerhof (PBE) functional was adopted for calculations of electron exchange‐correlation energy.^[^ ^47^, ^48^ ^]^ The cut‐off energy of 400 eV was applied for plane wave expansions set. The Brillouin zone was sampled with 2 × 2 × 2 Gama k‐point grids for geometric optimization of the slab surface cells. During the structural optimization, a vacuum spacing of 15 Å was set to avoid interactions among slabs. The two topmost surface layers were allowed to fully relax until the total energy variation was less than 10^−5^ eV and all forces on each atom were less than 0.02 eV Å^−1^. All calculations are spin polarized. For the HER process, NiCo_2_Se_4_ (001) and V_Se_‐NiCo_2_Se_4_ (001) were modeled by p (2 × 2) supercell slabs consists of six atomic layers. The hydrogen adsorption energy (ΔE_H*_) is calculated as follow: ΔE_H*_ = E_H*/slab_ − 1/2E_H2_ − E_slab_, where E_H*/slab_ is the total energy of the surface with an adsorbed H atom, E_H2_ and E_slab_ represent the energy of a H_2_ gas molecule and the bare surface, respectively. Additionally, the water adsorption energy (ΔE_H2O*_) can be also obtained by the following equation: ΔE_H2O*_ = E_H2O*/slab_ − E_H2O_ − E_slab_. The free energy of hydrogen adsorption (ΔG_H*_) can be calculated according to: ΔG_H*_ = ΔE_H*_ + ΔE_ZPE_ − TΔS_H_, where ΔE_H*_ is calculated total energy of H adsorption, ΔE_ZPE_ and ΔS_H_ are zero‐point energy change and entropy change, respectively. The reactant (H_2_O) and intermediates (OH and H) were performed to adsorbing on all possible active sites of the slab surfaces. The transition states (TS) for H_2_O dissociation were located using the climbing image‐nudged elastic band (CI‐NEB) method. The TS configurations were verified by vibration analyses with only one imaginary frequency.

For the OER process, NiCoOOH (001) and O_v_‐NiCoOOH (001) were modeled by p (2 × 2) supercell slabs with nine atomic layers. The following four‐step mechanism was adopted to describe the OER process:

(1) $$*+H_{2}O\to *\mathrm{OH}+H^{+}+e^{-}$$

(2) $$*\mathrm{OH}\to *O+H^{+}+e^{-}$$

(3) $$*O+H_{2}O\to *\mathrm{OOH}+H^{+}+e^{-}$$

(4) $$*\mathrm{OOH}\to *+O_{2}+H^{+}+e^{-}$$

The free‐energy change (ΔG) was estimated by the following equation:

(5) ΔG=ΔE+ΔZPE−TΔS−eU

where ΔE, ΔZPE, ΔS, and U are the energy change, changes of zero‐point energy, entropy changes and applied potential against RHE at standard conditions, respectively.

### COMSOL Multiphysics Simulations

Governing equations: The electric field and concentration distribution of ions were simulated by combining the “Electrostatics” and “Transport of Diluted Species” models using the COMSOL Multiphysics finite‐element based solver. The Poisson–Nerst–Planck equations were solved in the steady state. In this model, the diffusion coefficients of cation and anion were 2.14 e^−9^ and 7 e^−9^ m^2^ s^−1^, respectively. Besides, the electrolyte conductivity was assumed to be 2.5 S m^−1^.

The current density was simulated in the electrochemical module via Butler–Volmer equation. In detail, the anodic and cathodic charge transfer coefficients were both equal to 0.5. The temperature was taken to be 273.15 K. Based on the Arrhenius law, the exchange current density could be calculated by activation energy.

Computational domain: 2D continuum model was built in this work to represent the 3D structures of electrodes. All sizes were shown in the relative figures.

Mesh: Triangular meshes with normal size were used for all simulations. On the electrode surface, the size was with the range of 0.01∼0.5 nm. While for other parts, the elements sizes increased to 4 nm.

Boundary conditions: The ion concentrations for both cation and anion were set to 1 mol L^−1^ at the electrode boundary.

## Conflict of Interest

The authors declare no conflict of interest.

## Supporting information

Supporting Information

## Data Availability

The data that support the findings of this study are available from the corresponding author upon reasonable request.
