# Supplementary material for: Hierarchical NiCo2Se4 Arrays Composed of Atomically Thin Nanosheets: Simultaneous Improvements in Thermodynamics and Kinetics for Electrocatalytic Water Splitting in Neutral Media
Source: Adv Sci (Weinh). 2024 Jun 18;11(31):2402889. doi: 10.1002/advs.202402889 (PMC11336961; doi:10.1002/advs.202402889)
Supplement: Supplementary file 1 — Supporting Information [file ADVS-11-2402889-s001.docx]

**Electronic Supplementary Material**

**Hierarchical NiCo_2_Se_4_ Arrays Composed of Atomically Thin Nanosheets: Simultaneous Improvements in Thermodynamics and Kinetics for Electrocatalytic Water Splitting in Neutral Media**

Hongyu Chen*^a^*§, Yongsheng Xu*^b^*§, Xiaojie Li *^c^*, Qing Ma*^a^*, Delong Xie*^a^*, Yi Mei*^a^*, Guojing Wang*^a^** and Yuanzhi Zhu*^a^**

*^a^* Faculty of Chemical Engineering, Yunnan Provincial Key Laboratory of Energy Saving in Phosphorus Chemical Engineering and New Phosphorus Materials, Kunming University of Science and Technology, Kunming 650500, Yunnan, China.

*^b^* School of Chemistry and Chemical Engineering/State Key Laboratory Incubation Base for Green Processing of Chemical Engineering, Shihezi University, Shihezi 832000, China.

*^c^* PetroChina Shenzhen New Energy Research Institute.

E-mail: gjwang@kust.edu.cn; yuanzhi_zhu@kust.edu.cn

§ The authors contributed equally to this work.

**Experimental Section**

**Materials.** Nickel(II) nitrate hexahydrate (Ni(NO_3_)_2_·6H_2_O, 98%), Cobalt(II) nitrate hexahydrate (Co(NO_3_)_2_·6H_2_O, ≥98%), urea (NH_2_CONH_2_, 99%), sodium borohydride (NaBH_4_, 98%), potassium dihydrogen phosphate (KH_2_PO_4_, ≥99.5%), dipotassium hydrogen phosphate (K_2_HPO_4_, ≥99%), acetone (CH_3_COCH_3_, 99%) and nitric acid (HNO_3_, 68–70%) were purchased from Sinopharm Chemical Reagent Co., Ltd.. Selenium powder (99.9%, 200 mesh) was obtained from Aladdin Chemistry Co., Ltd.. Carbon fiber paper (CFP, ~180 μm in thickness) were ordered from Wuhan Cetech Co., Ltd.. All reagents were used without any further purification. Deionized water (18.2 MΩ) produced from a Milli-Q purification system was used for the samples synthesis and electrochemical measurements.

**Characterization of materials**

The X-ray diffraction (XRD) patterns were recorded using a Rigaku SmartLab diffractometer equipped with a Cu Kα X-ray source (λ = 1.5406 Å, generated at 40 kV and 100 mA), operating at a scanning rate of 0.06° s^−1^ in the 2θ range from 10° to 90°. Scanning electron microscopy (SEM) images were obtained using a Hitachi SU-8010 field-emission scanning electron microscope operating at 5 kV. Transmission electron microscopy (TEM), High-resolution TEM (HRTEM), scanning TEM (STEM), energy-dispersive X-ray spectroscopy (EDX) elemental mapping images and EDX were acquired using an FEI Talos F200S transmission electron microscope accompanied by two energy disperse X-ray spectrometers (Super-EDS) operated at 200 kV. X-ray photoelectron spectroscopy (XPS) measurements were conducted using a ESCALAB 250Xi spectrometer with a hemispherical energy analyzer, employing a monochromatized microfocused Al-Kα (hv = 1486.58 eV) X-ray source. The binding energies (BEs) of the core levels were calibrated by setting the adventitious C1s peak at 284.8 eV. Electron paramagnetic resonance (EPR) dates were obtained on a Bruker EMXplus. Inductively coupled plasma atomic emission spectrometry (ICP-AES) measurements was performed on Thermo Scientific iCAP 6300 after dissolving the sample in aqua regia. X-ray Absorption Fine Structure (XAFS) spectra at Ni and Co K-edge were measured in transmission mode on the 06ID superconducting wiggler sourced hard X-ray microanalysis beamline Beijing Synchrotron Radiation Facility. The XAFS raw data were processed and analyzed by Athena and Artemis program packages from the Demeter software. For Wavelet Transform analysis, the χ(k) exported from Athena was imported into the Hama Fortran code.

**Adsorbed cation/anion Measurement**

The ability of the sample to absorb cation/anion was characterized by Inductively coupled plasma atomic emission spectrometry (ICP-AES). Placing the sample electrode in 1 M PBS with a constant potential at -0.1 V (for HER) and 1.5 V (for OER) for 120 s, respectively, and then remove into 10 mL ultrapure water and shaking it for 20 s, repeating the above steps 10 times to capture the K^+^/H^+^ or H_2_PO_4_^−^/OH^−^ adsorbed on the surface of the electrode. The electrode in 1 M PBS without voltage was used as a blank background. Finally, the concentration of K^+^/H^+^ or H_2_PO_4_^−^/OH^−^ in ultrapure water after the experiment with deducted the background represents the ability of the electrode surface to absorb cation/anion. The detailed values during the process of the test were shown in Table S5.


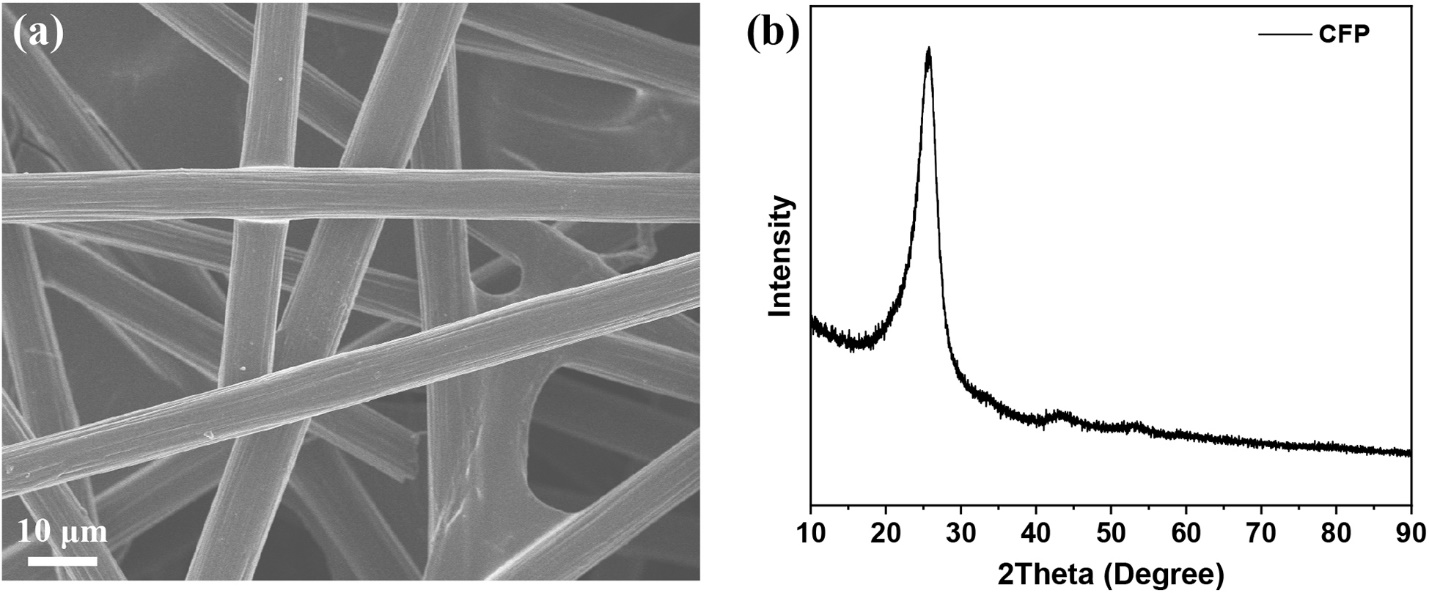


**Figure S1.** (a) SEM image and (b) XRD pattern of Carbon fiber paper (CFP).


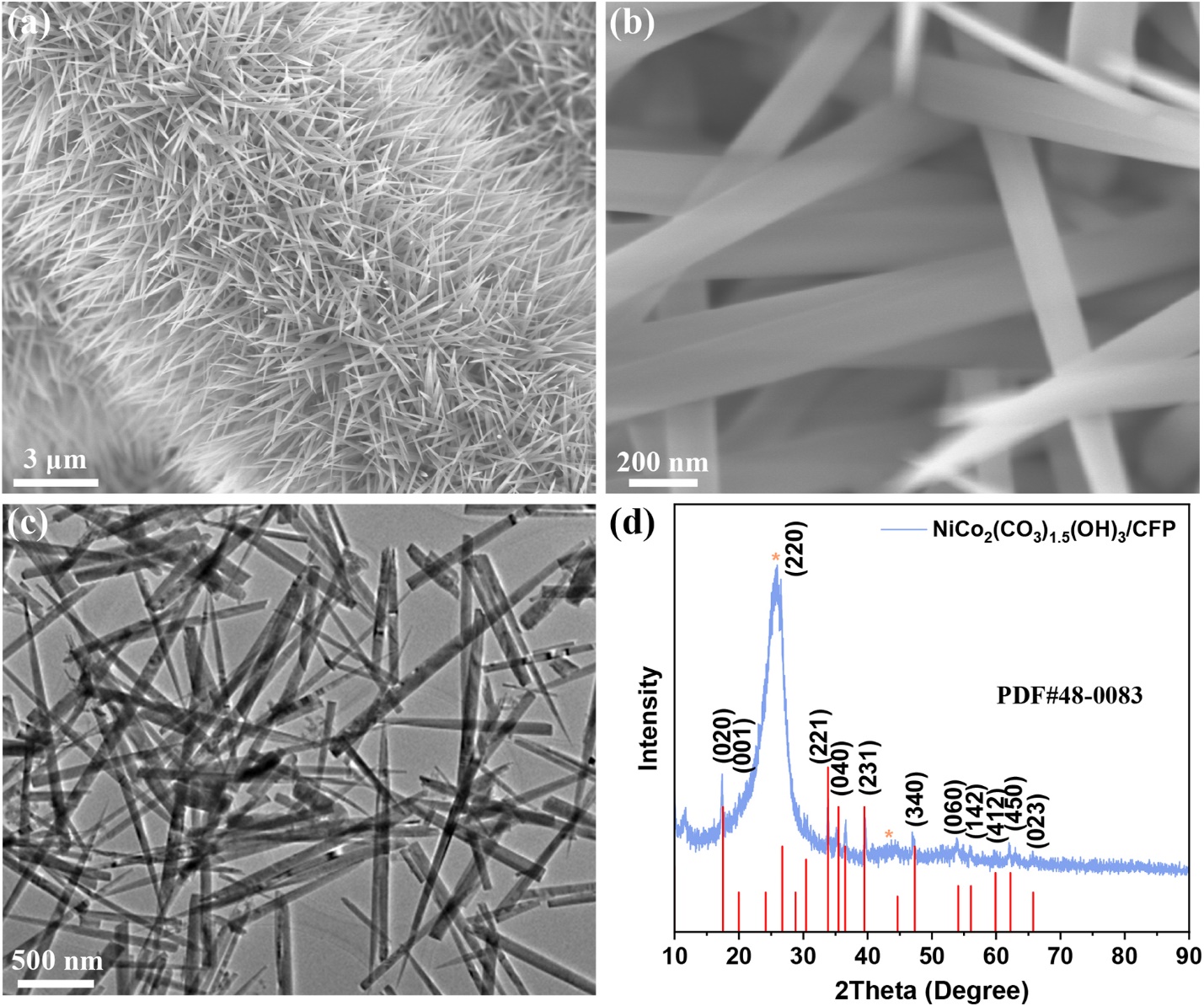


**Figure S2.** (a, b) SEM, (c) TEM images and (d) XRD pattern of the NiCo_2_(CO_3_)_1.5_(OH)_3_ NNs/CFP.


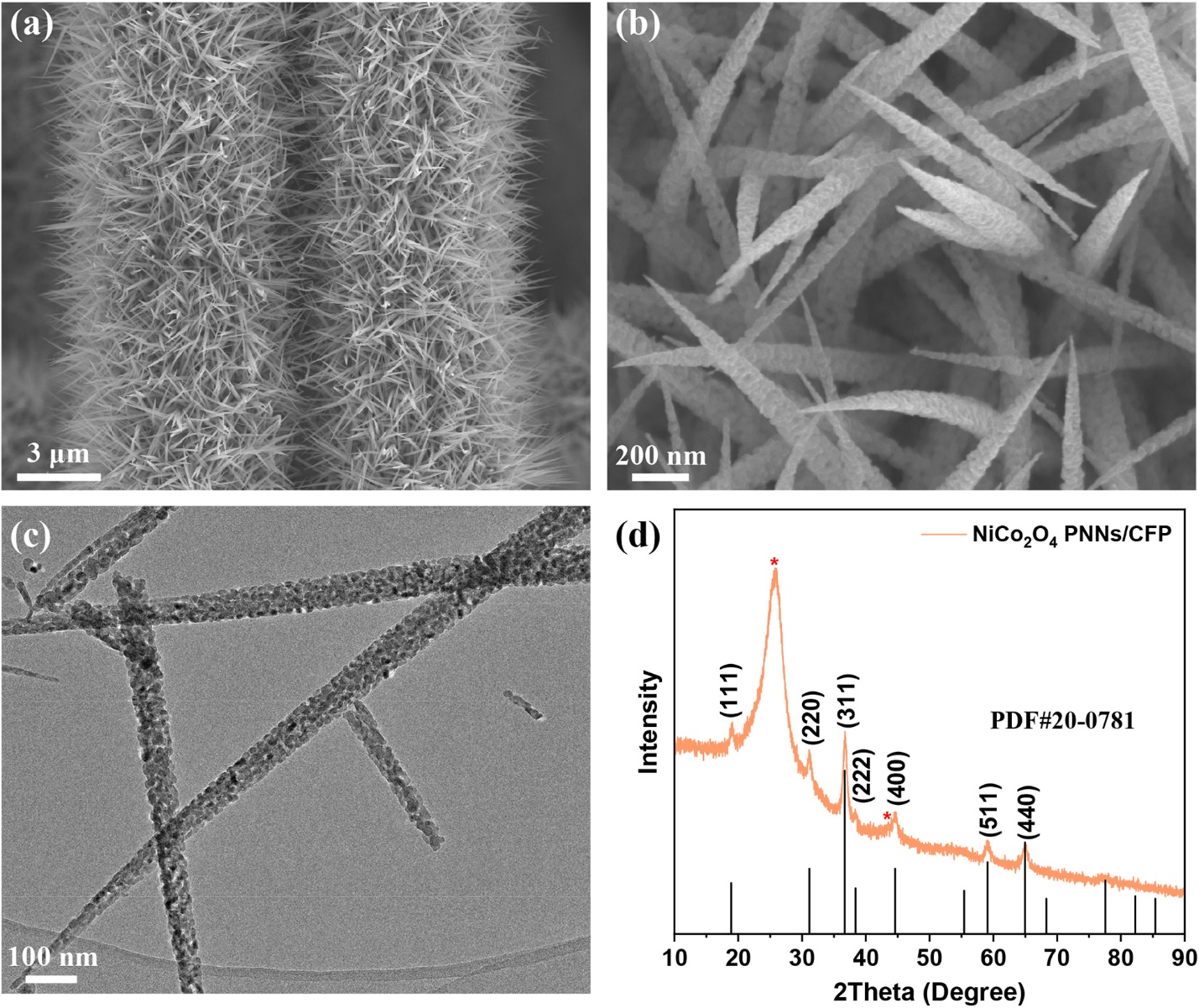


**Figure S3.** (a, b) SEM, (c) TEM images and (d) XRD pattern of the NiCo_2_O_4_ PNNs/CFP.


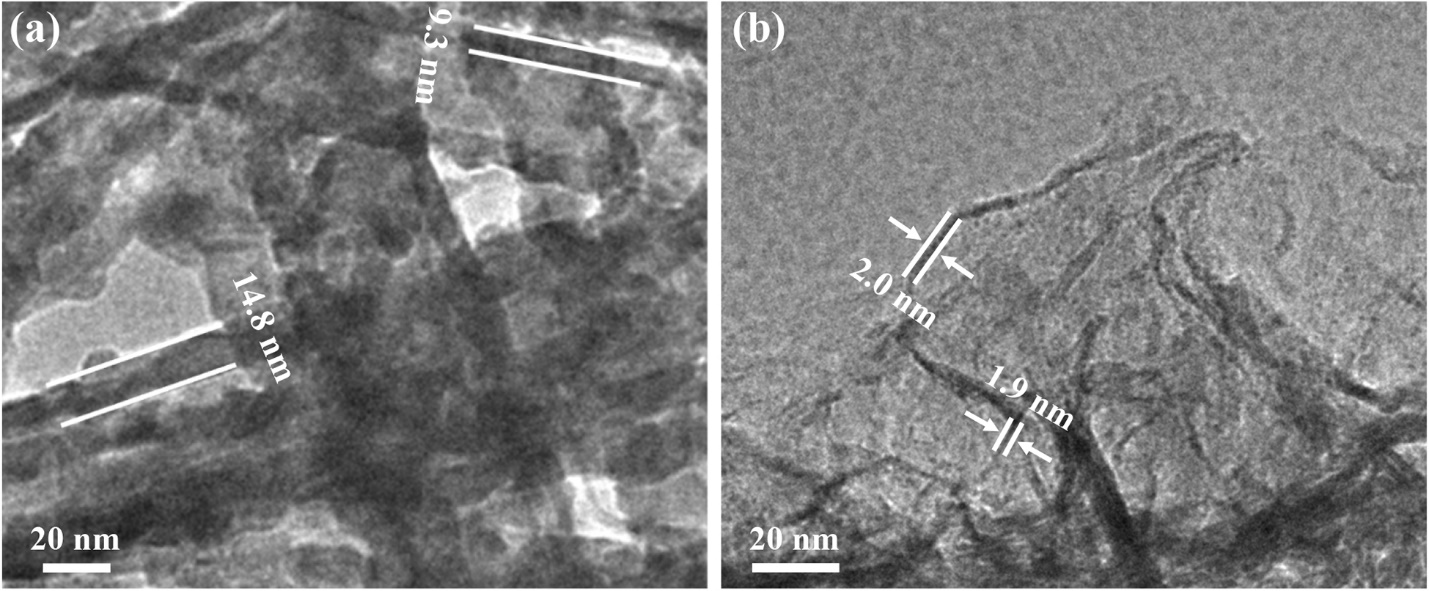


**Figure S4.** The TEM images of (a) NiCo_2_Se_4_ HNNs/CFP and (b) NiCo_2_Se_4_ HUNSs/CFP.


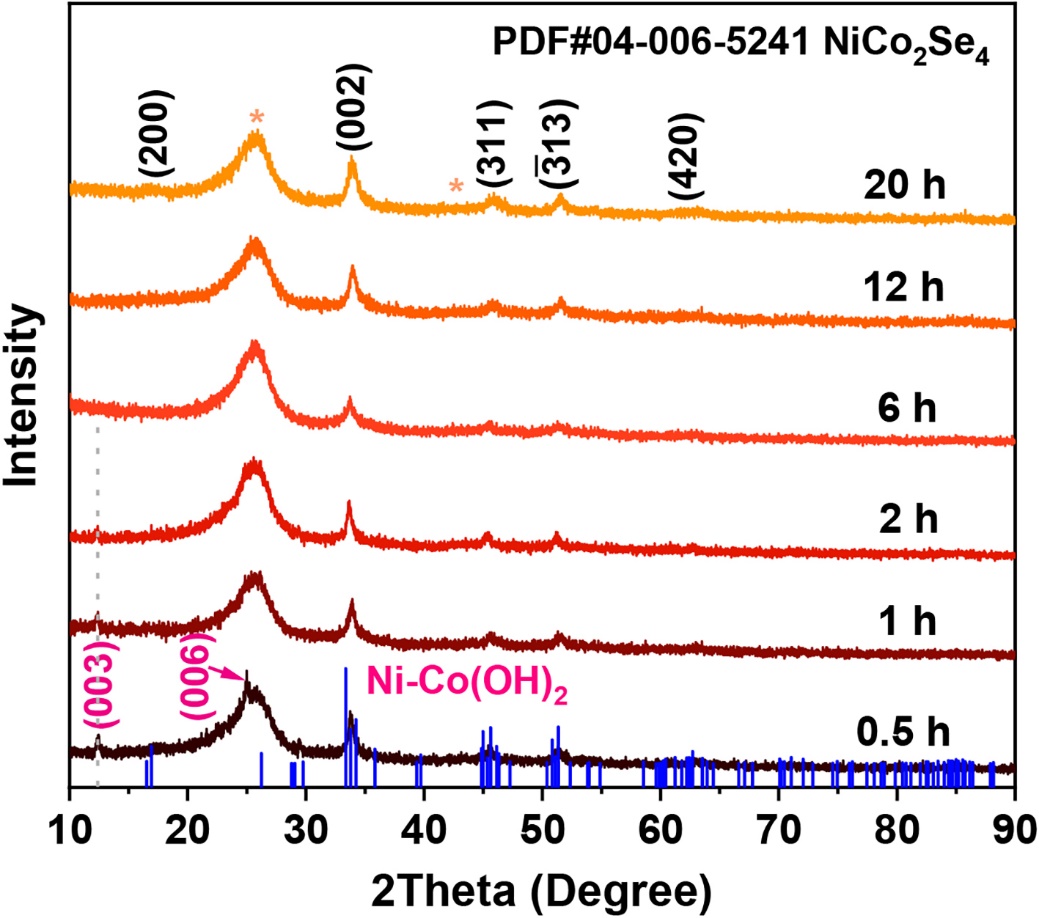


**Figure S5.** The XRD patterns of NiCo_2_O_4_ PNNs under different selenization times (0.5h, 1h, 2h, 6h, 12h, 20h) for 120 ℃.


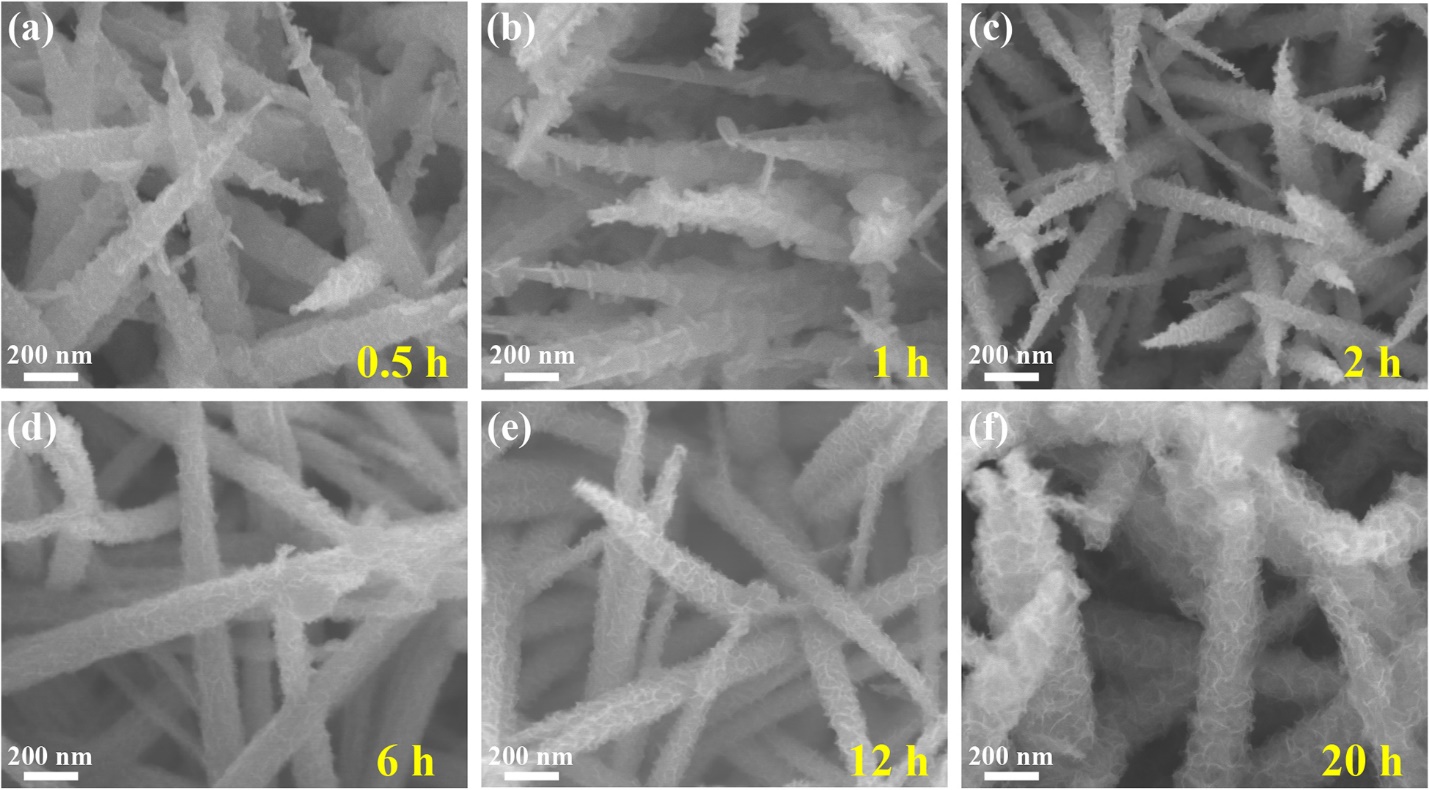


**Figure S6.** The SEM images of NiCo_2_O_4_ PNNs under different selenization times (0.5h, 1h, 2h, 6h, 12h, 20h) for 120 ℃.


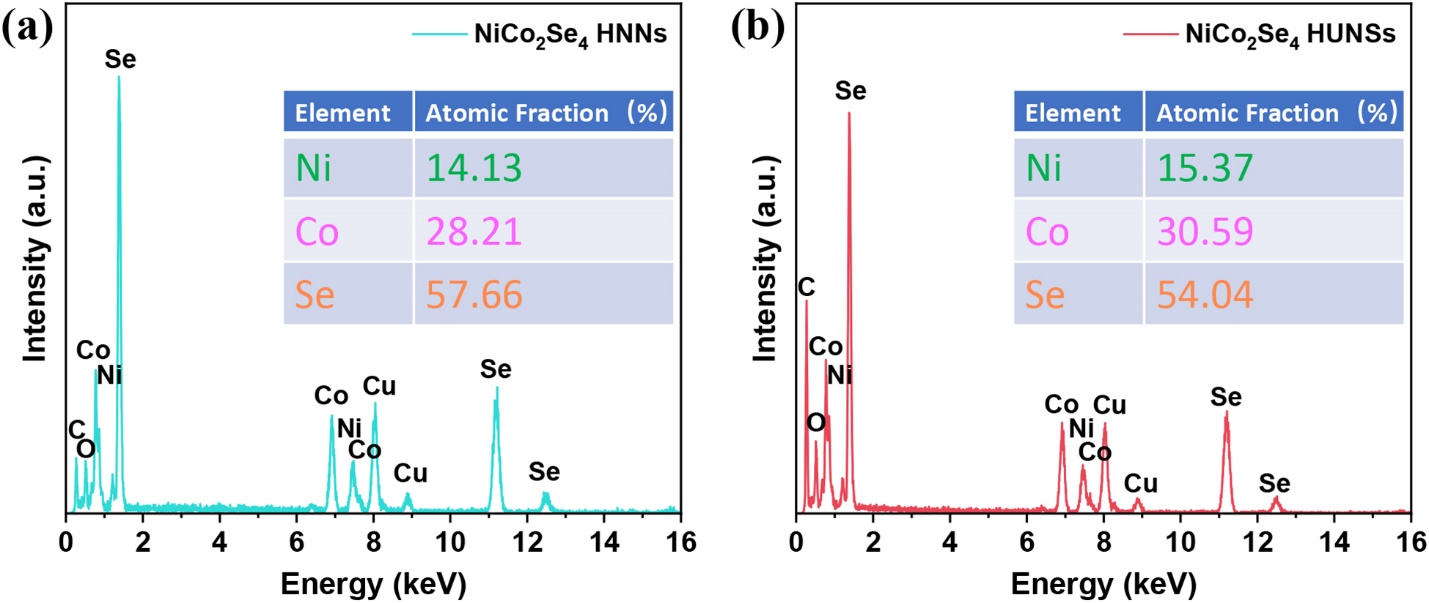


**Figure S7.** EDX spectra of the (a) NiCo_2_Se_4_ HNNs/CFP and (b) NiCo_2_Se_4_ HUNSs/CFP.

**
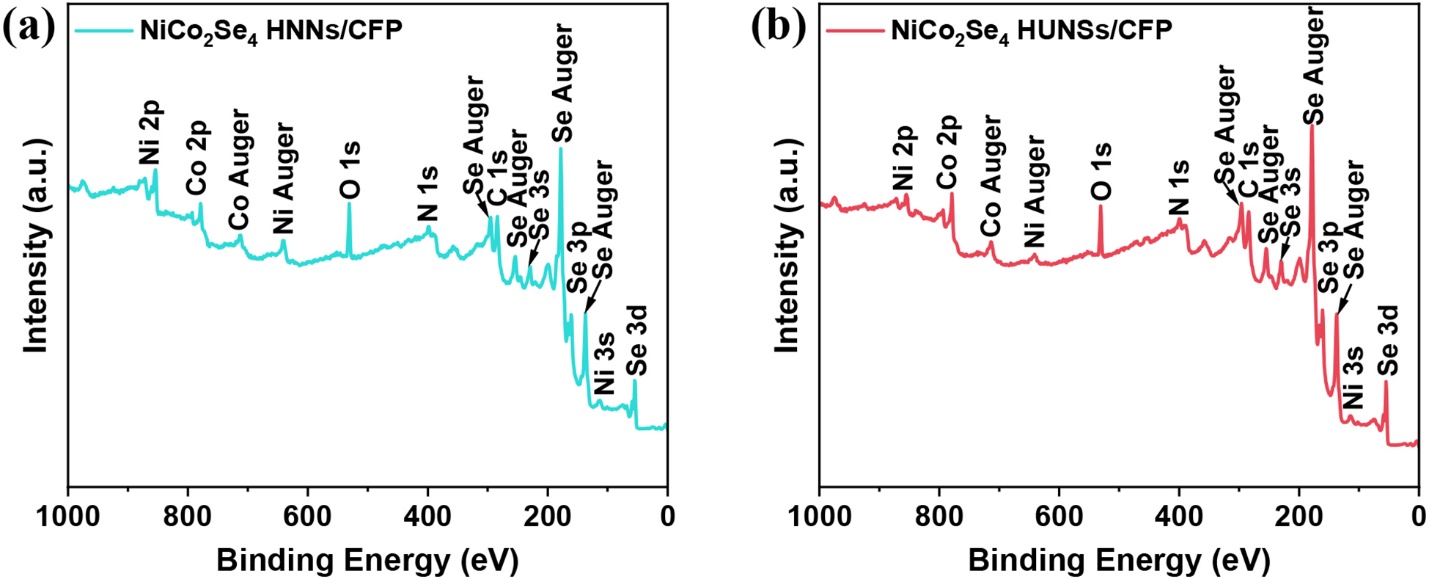
**

**Figure S8.** The XPS survey spectrum of (a) NiCo_2_Se_4_ HNNs/CFP and (b) NiCo_2_Se_4_ HUNSs/CFP.


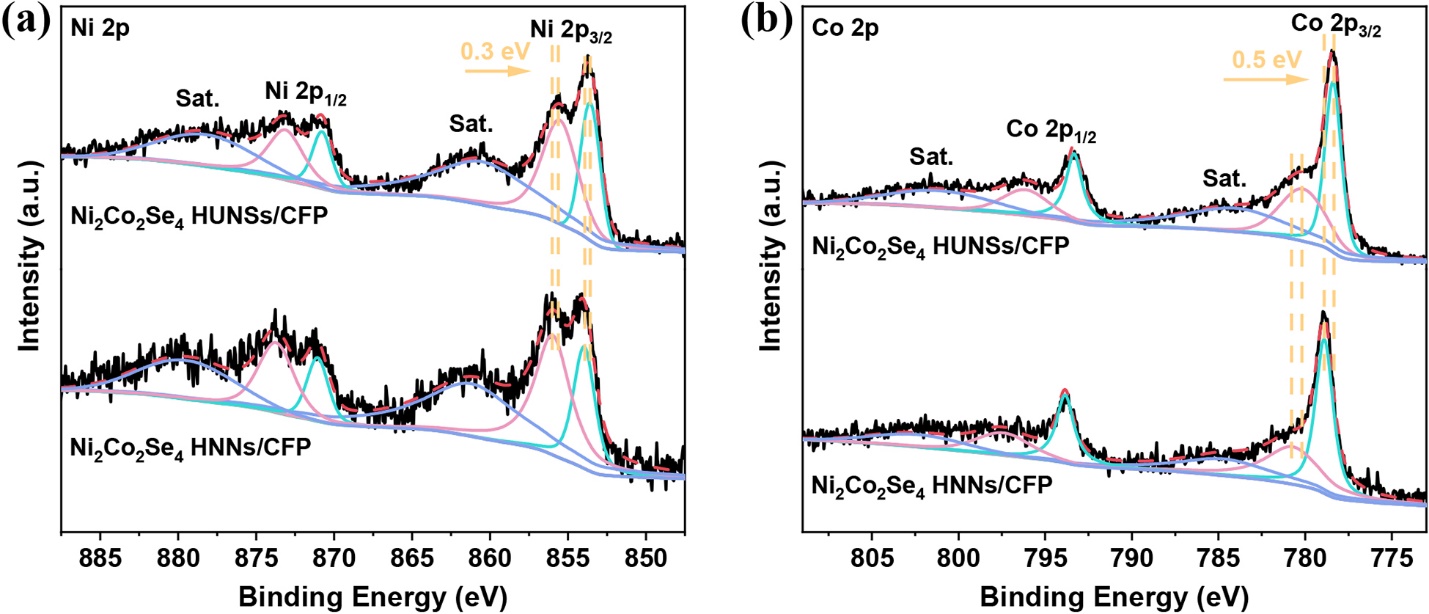


**Figure S9.** High-resolution (a) Ni 2p and (b) Co 2p XPS spectra of NiCo_2_Se_4_ HNNs/CFP and NiCo_2_Se_4_ HUNSs/CFP.


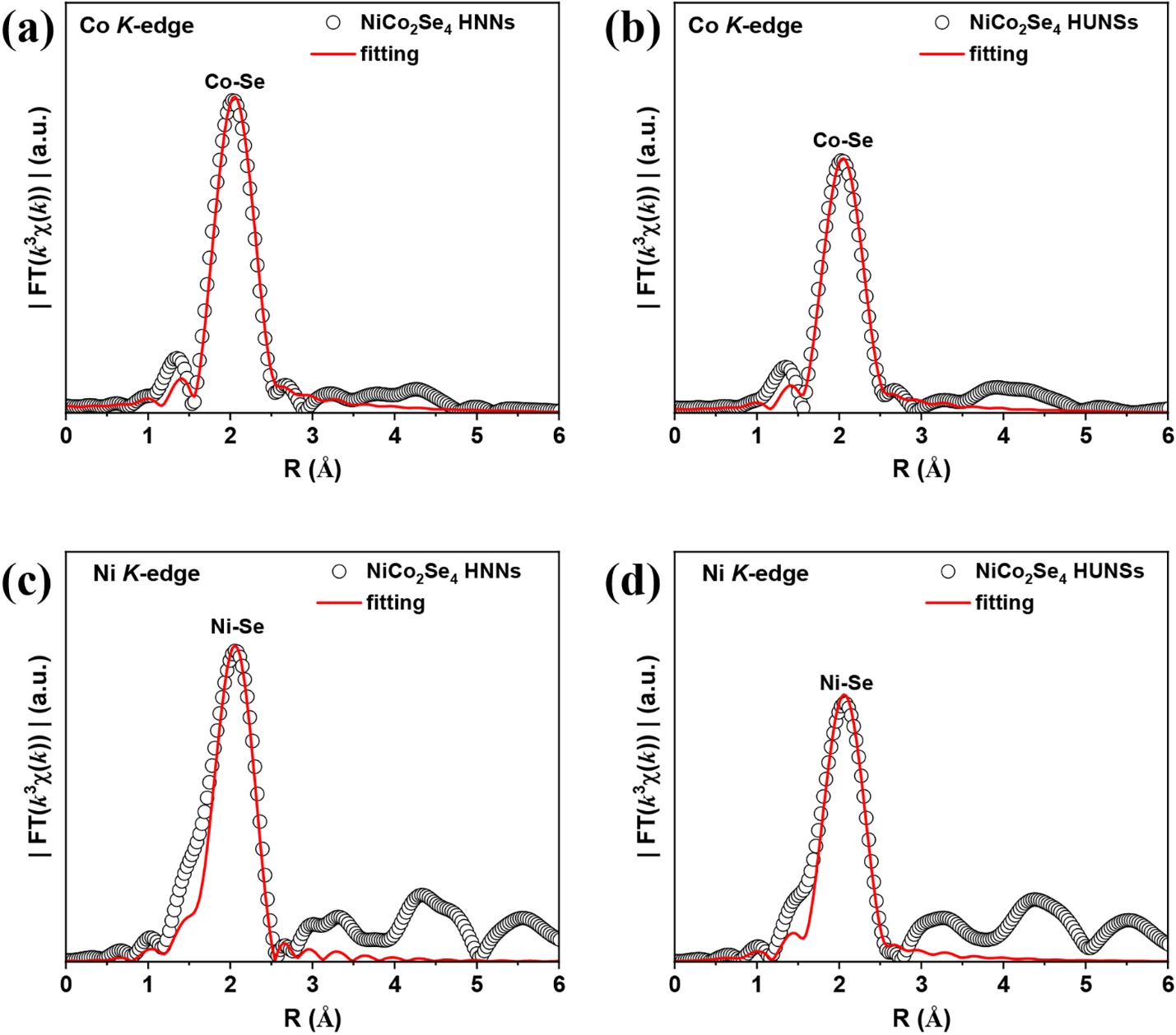


**Figure S10.** EXAFS fitting in R space at the (a, b) Co K-edge and (c, d) Ni K-dege of (a, c) NiCo_2_Se_4_ HNNs and (b, d) NiCo_2_Se_4_ HUNSs.


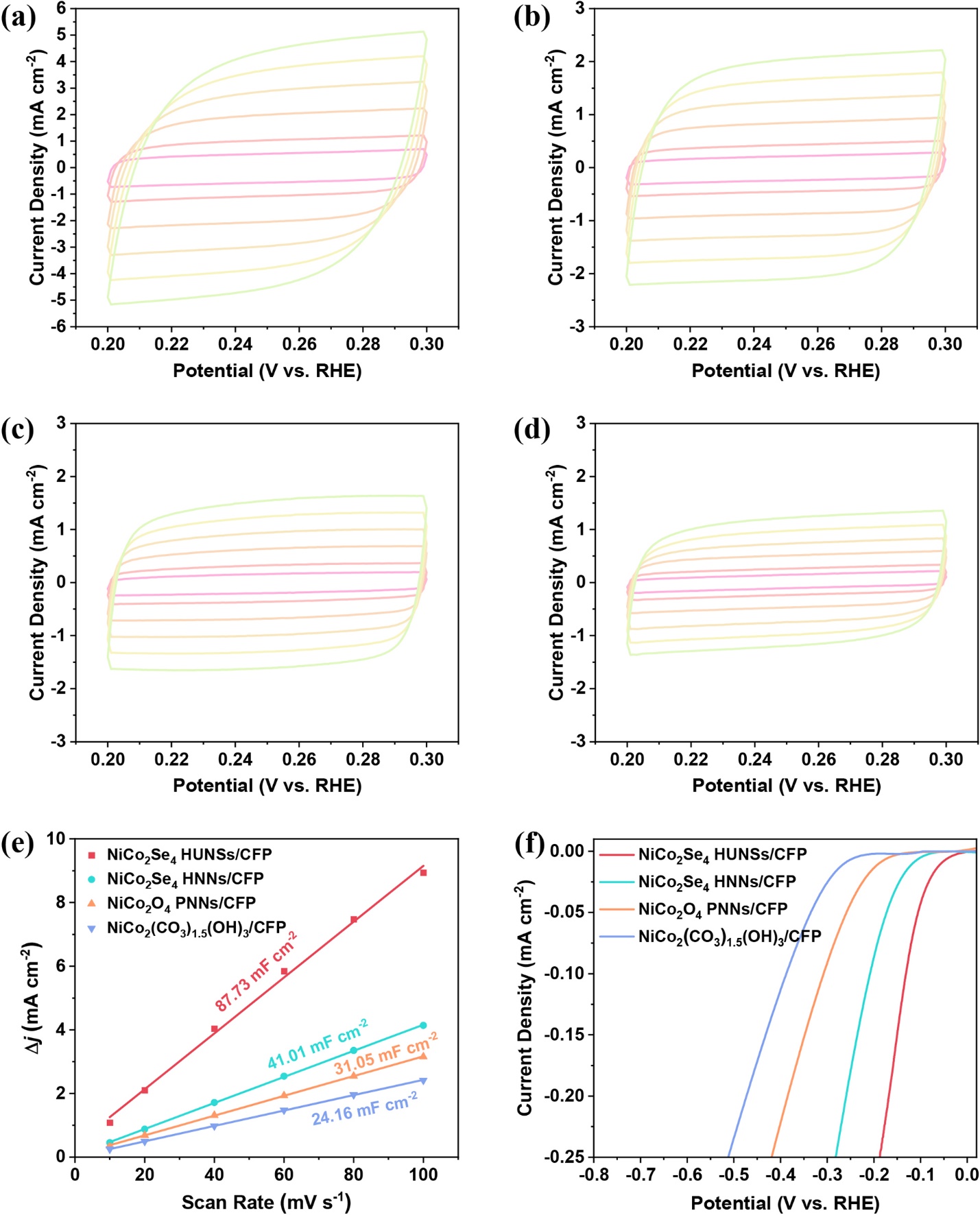


**Figure S11.** Cyclic voltammograms of the (a) NiCo_2_Se_4_ HUNSs/CFP, (b) NiCo_2_Se_4_ HNNs/CFP, (c) NiCo_2_O_4_ PNNs/CFP and (d) NiCo_2_(CO_3_)_1.5_(OH)_3_ NNs/CFP electrodes, which are used to estimate the double layer capacitances (C_dl_). Sweep rates at 10, 20, 40, 60, 80 and 100 mV s^−1^ were chosen. (e) capacitance current vs. v plots, (f) Specific activity based on ECSA of the various catalyst samples.


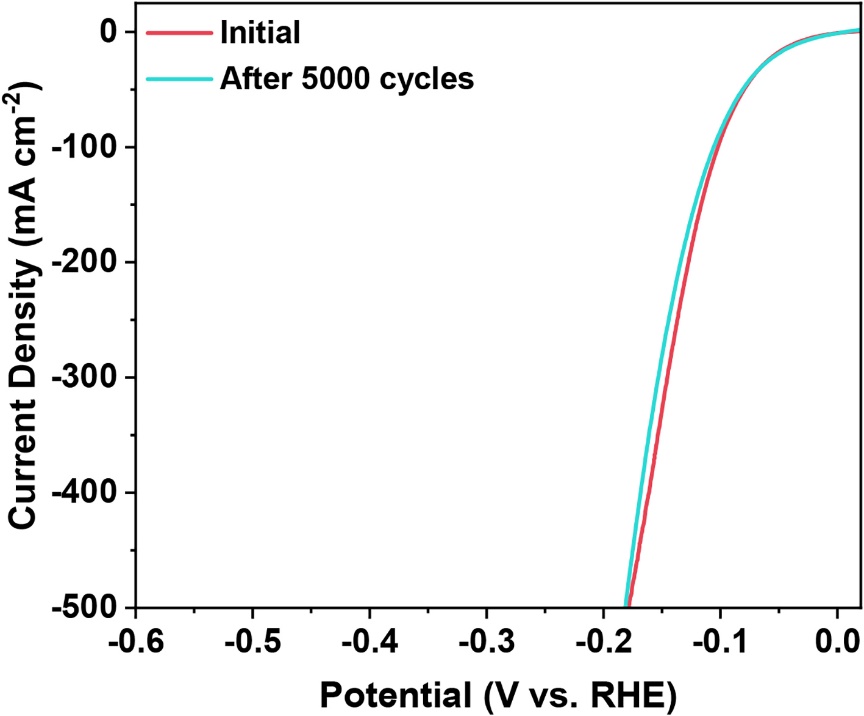


**Figure S12.** Polarization curves of NiCo_2_Se_4_ HUNSs/CFP corresponding to the 1st and 5000th potential cycle recorded in 1 M PBS.

**
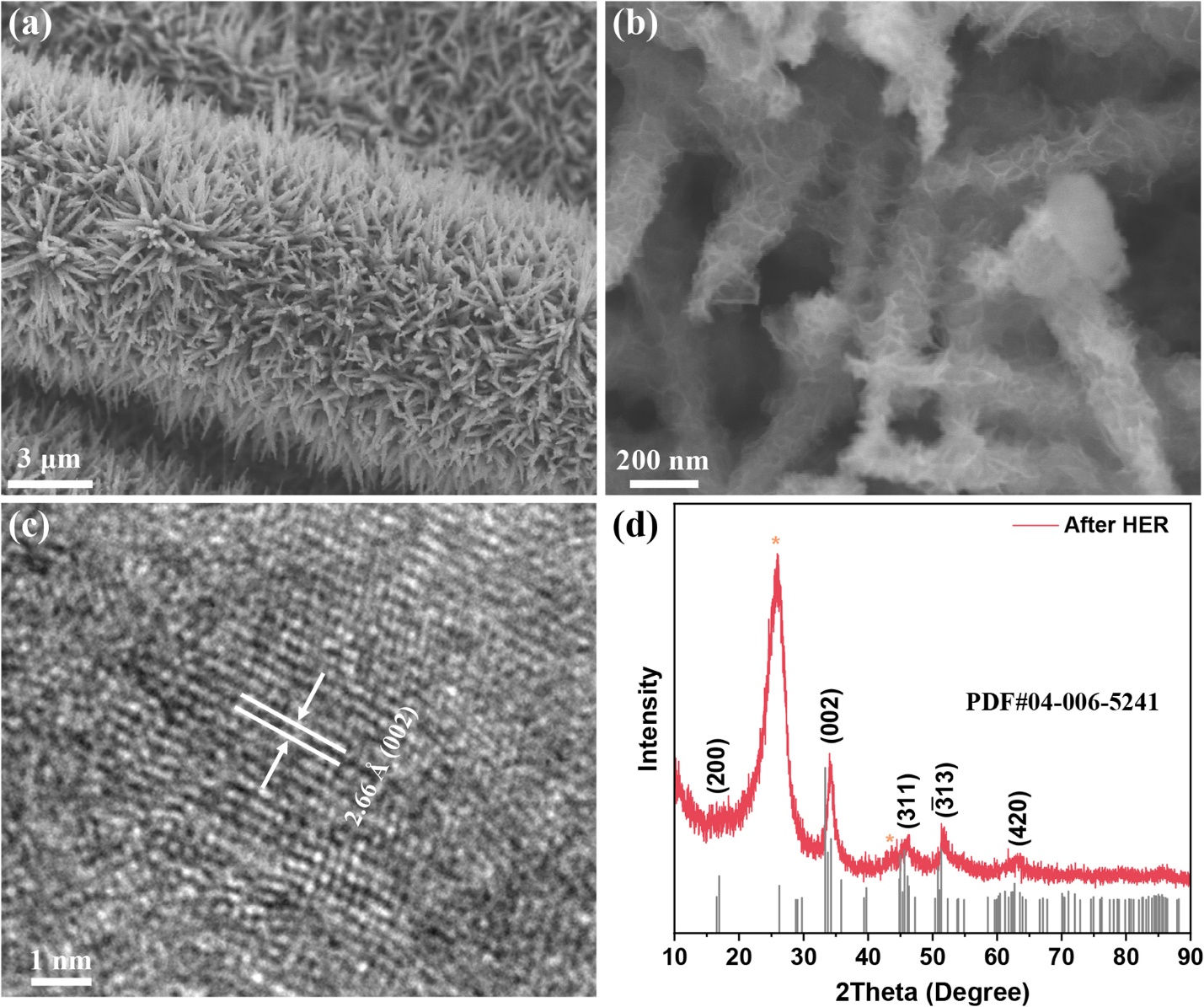
**

**Figure S13.** (a, b) SEM, (c) TEM images and (d) XRD pattern of the NiCo_2_Se_4_ HUNSs/CFP after a stability test of HER for 50 h.


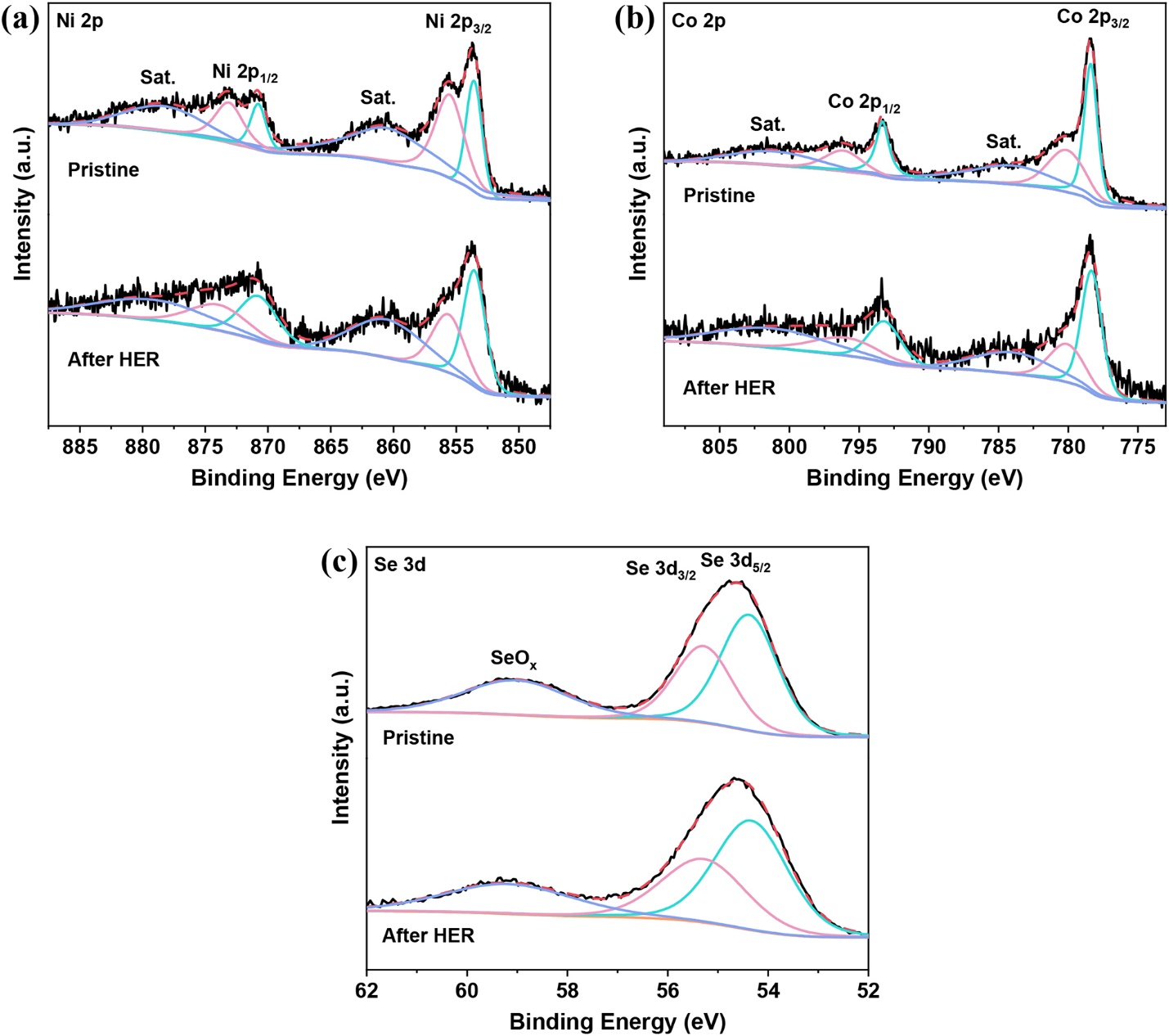


**Figure S14.** High-resolution (a) Ni 2p, (b) Co 2p and (c) Se 3d XPS spectra of the NiCo_2_Se_4_ HUNSs/CFP before and after a stability test of HER for 50 h.


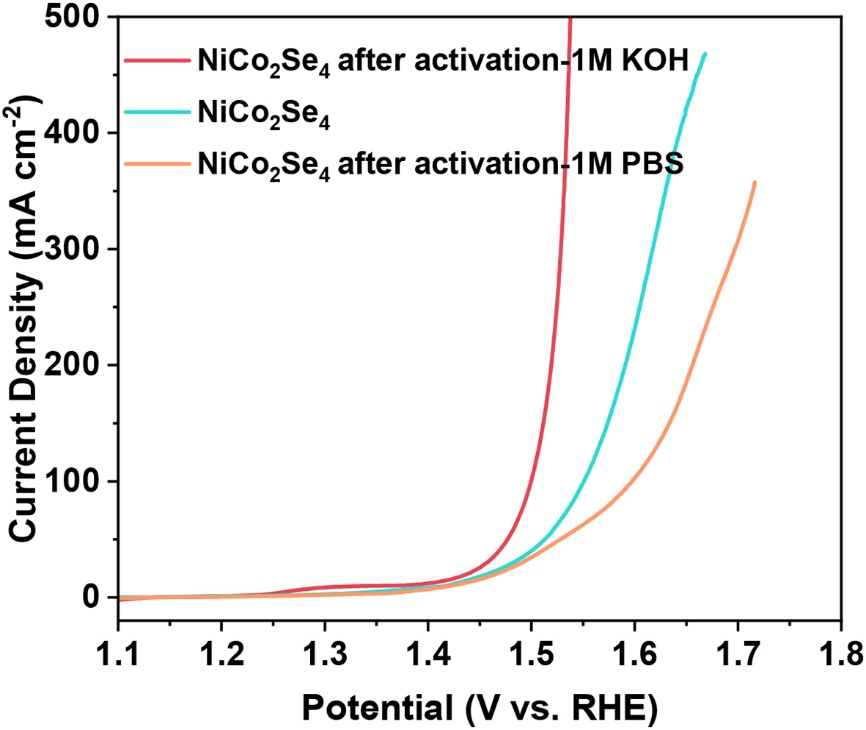


**Figure S15.** Polarization curves of the NiCo_2_Se_4_ HUNSs/CFP after alkaline activation, NiCo_2_Se_4_ HUNSs/CFP after neutral activation and pristine NiCo_2_Se_4_ HUNSs/CFP measured in 1 M PBS. The observed oxidation peak at 1.3 V in 1 M PBS is attributed to the oxidation of Ni or Co to a higher valence state during the OER. In 1 M KOH, the NiCo_2_Se_4_ undergo more complete structural reconstruction, resulting in a greater number of exposed active sites and thus a more pronounced oxidation peak. The alkaline activation of NiCo_2_Se_4_ HUNSs/CFP was performed by a chronopotentiometry experiment at a constant current density of 10 mA cm^−2^ for 10 h in 1 M KOH. Similarly, the neutral activation of NiCo_2_Se_4_ HUNSs/CFP was performed by the same process as above in 1 M PBS.


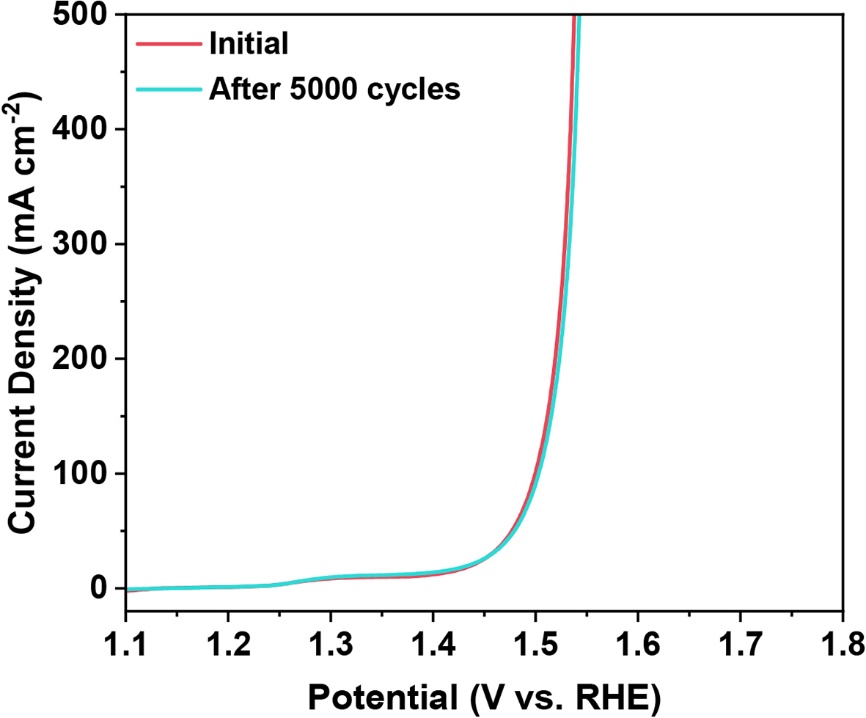


**Figure S16.** Polarization curves of NiCoOOH HUNSs/CFP corresponding to the 1st and 5000th potential cycle recorded in 1 M PBS.


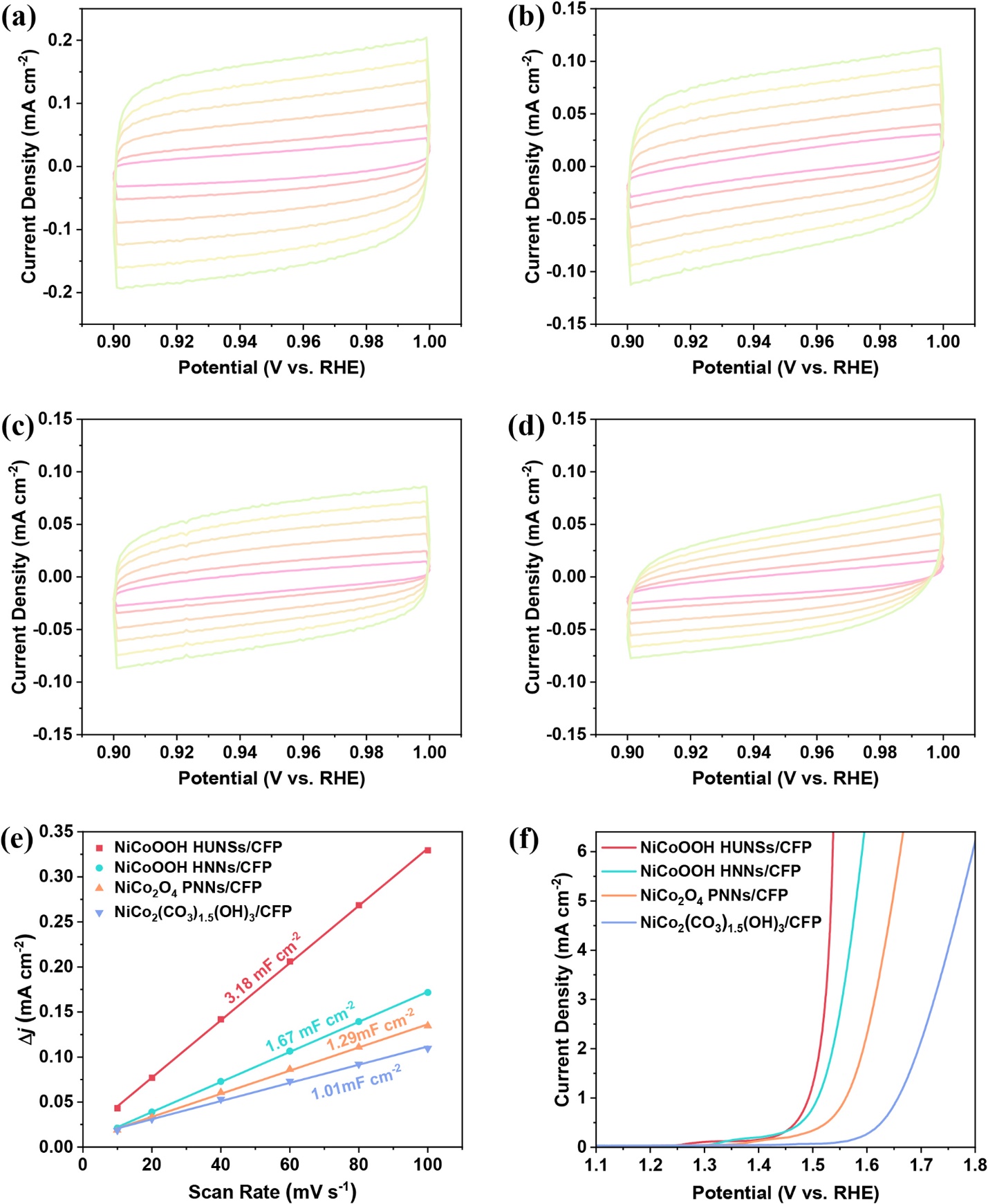


**Figure S17.** Cyclic voltammograms of the (a) NiCoOOH HUNSs/CFP, (b) NiCoOOH HNNs/CFP, (c) NiCo_2_O_4_ PNNs/CFP and (d) NiCo_2_(CO_3_)_1.5_(OH)_3_ NNs/CFP electrodes, which are used to estimate the double layer capacitances (C_dl_). Sweep rates at 10, 20, 40, 60, 80 and 100 mV s^−1^ were chosen. (e) capacitance current vs. v plots, (f) Specific activity based on ECSA of the various catalyst samples.

**
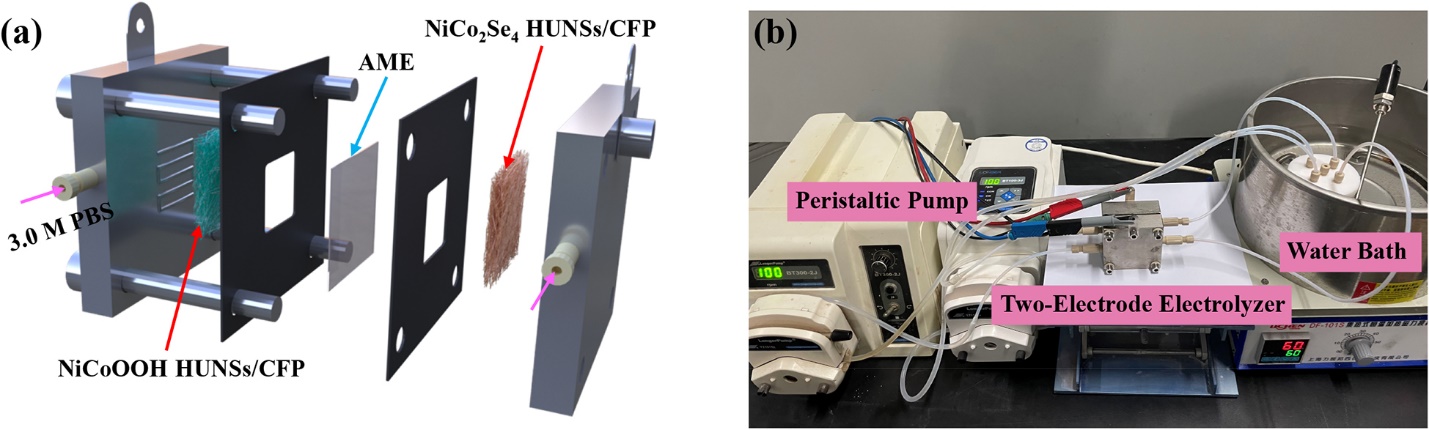
**

**Figure S18.** (a) Schematic diagram of AEM with NiCo_2_Se_4_ HUNSs/CFP||NiCoOOH HUNSs/CFP water splitting. (b) A photograph showing the experimental apparatus for the two-electrode electrolyzer flow-cell.


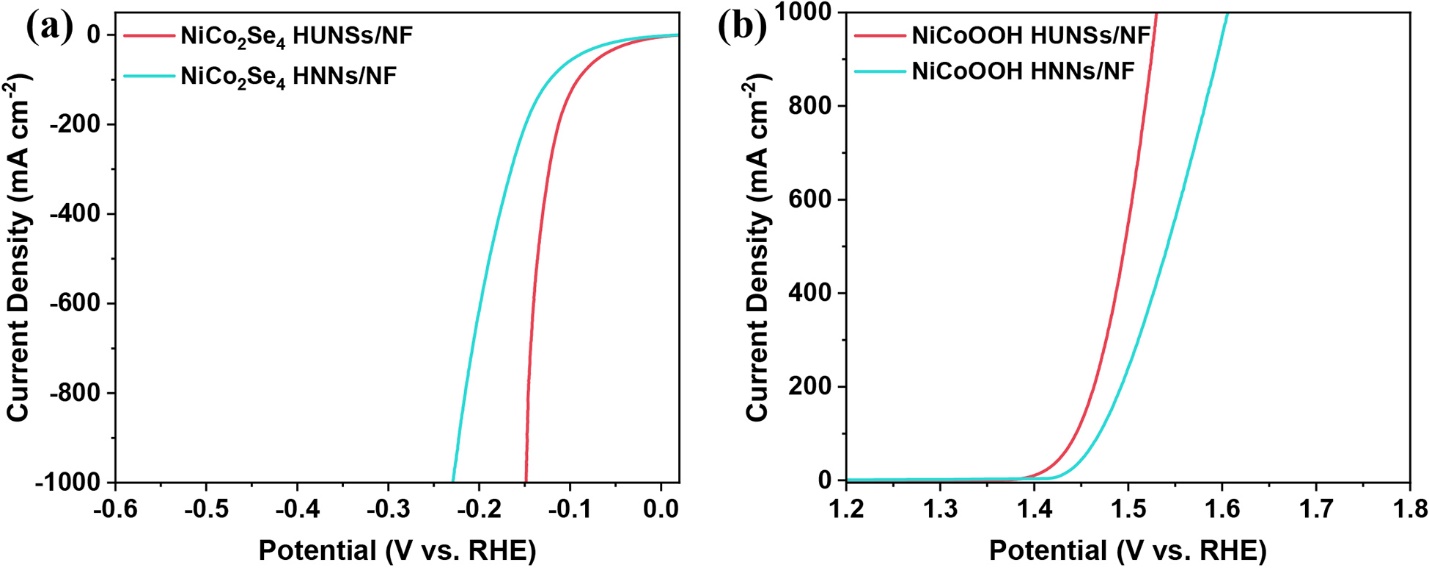


**Figure S19.** Polarization curves of (a) NiCo_2_Se_4_ HNNs/NF and NiCo_2_Se_4_ HUNSs/NF for HER and (b) NiCoOOH HNNs/NF and NiCoOOH HUNSs/NF for OER measured in 1 M PBS.

**
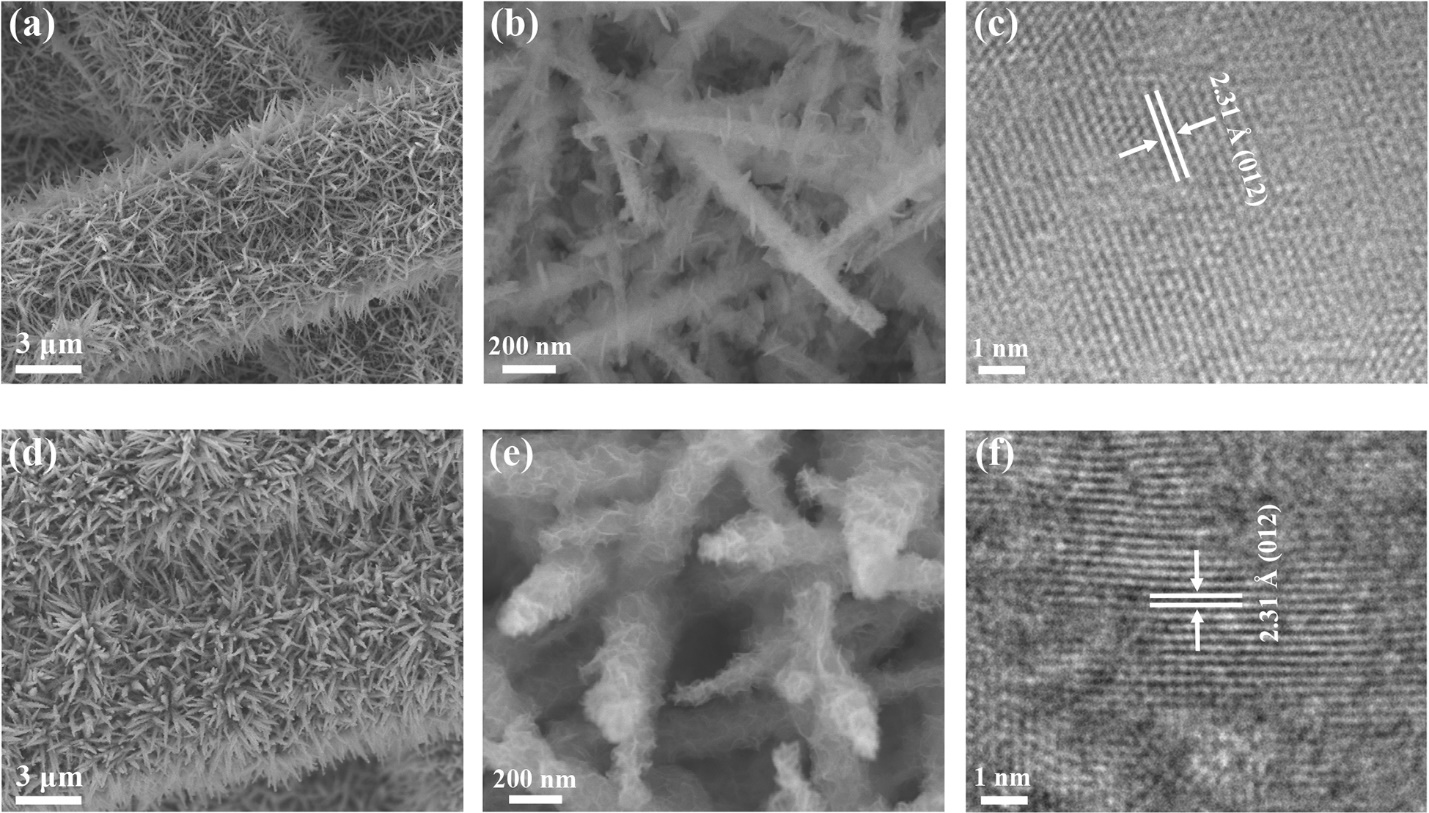
**

**Figure S20.** (a, d) Low-, and (b, e) high-magnification SEM, and (c, f) TEM images of (a-c) NiCoOOH HNNs/CFP and (d-f) NiCoOOH HUNSs/CFP.


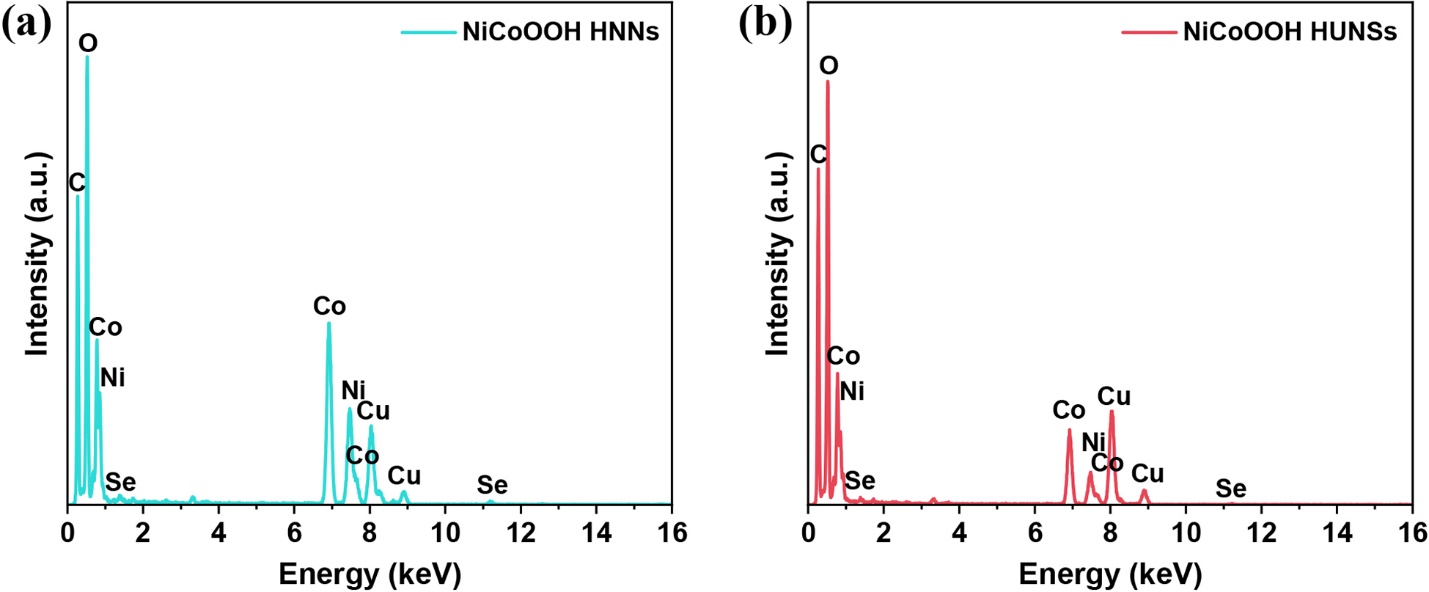


**Figure S21.** EDX spectra of the (a) NiCoOOH HNNs/CFP and (b) NiCoOOH HUNSs/CFP.


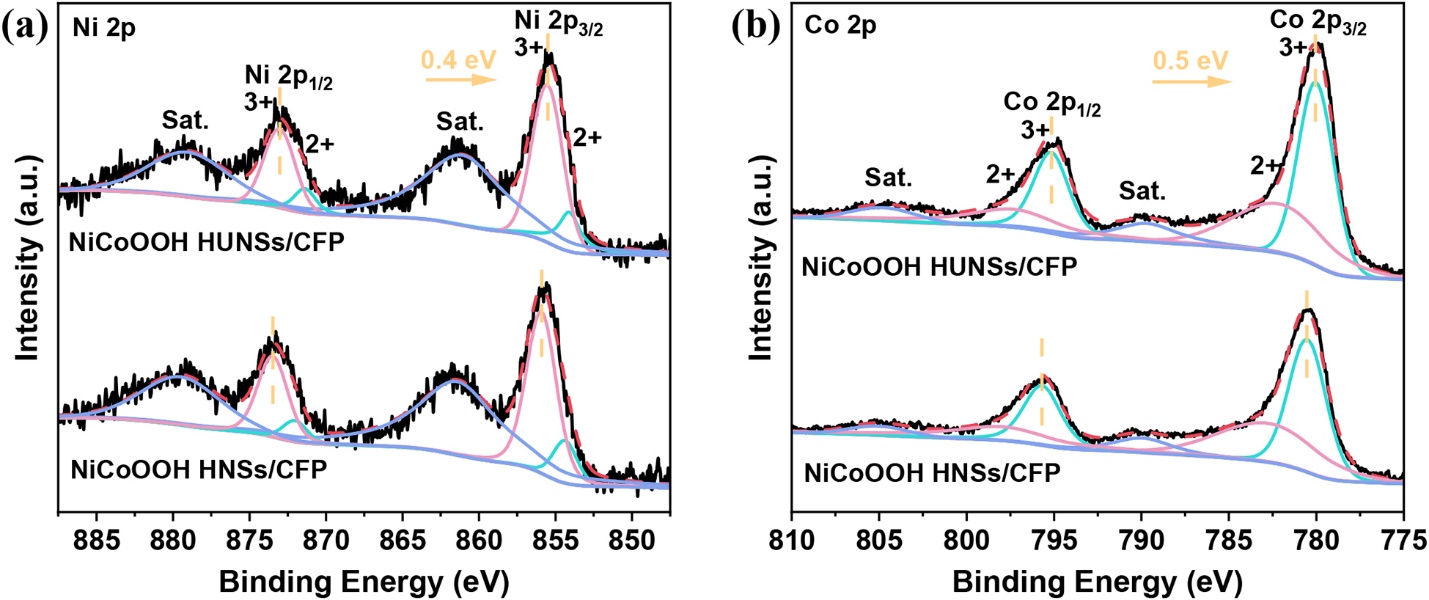


**Figure S22.** High-resolution (a) Ni 2p and (b) Co 2p XPS spectra of NiCoOOH HNNs/CFP and NiCoOOH HUNSs/CFP.

**
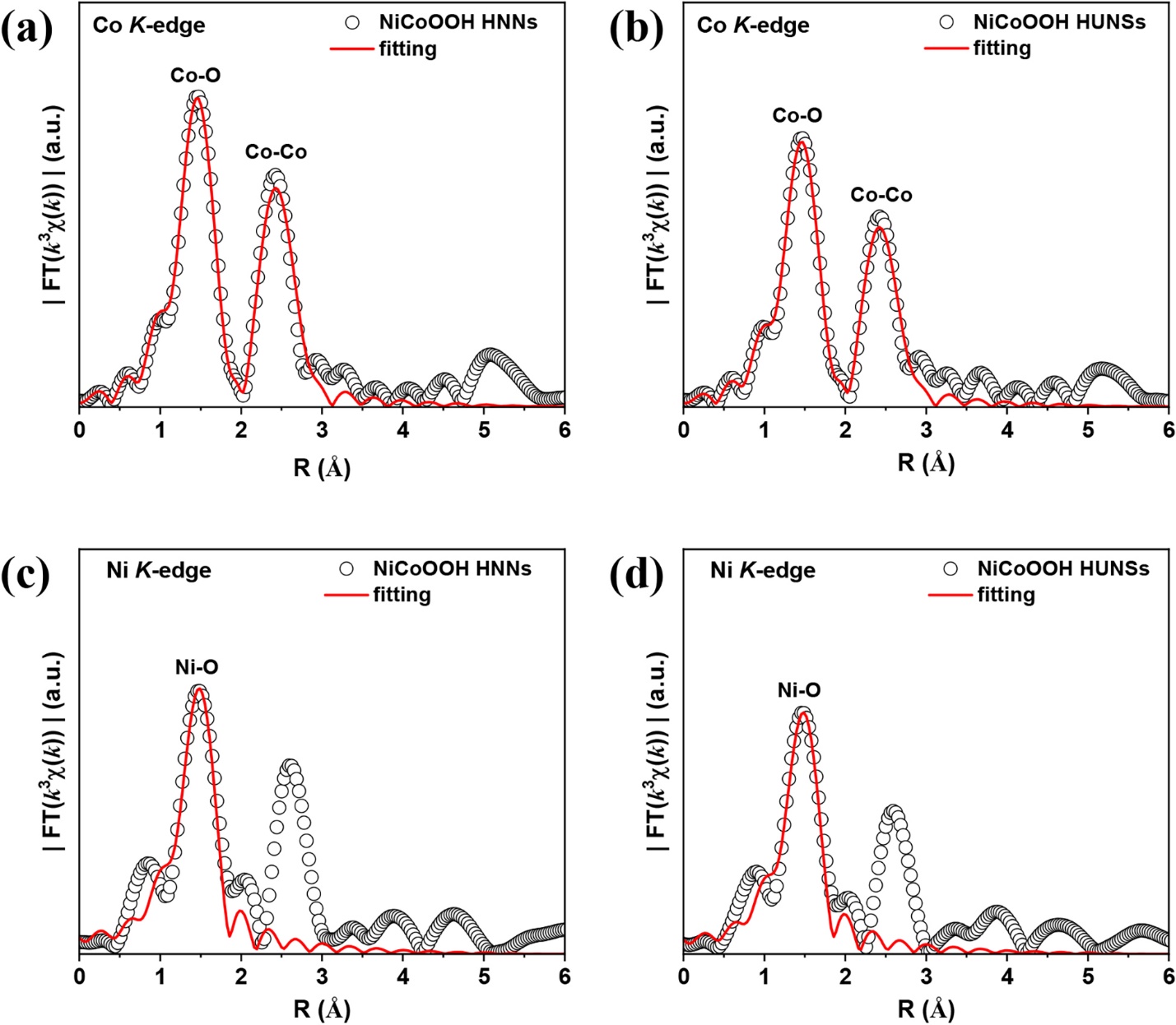
**

**Figure S23.** EXAFS fitting in R space at the (a, b) Co K-edge and (c, d) Ni K-dege of (a, c) NiCoOOH HNNs and (b, d) NiCoOOH HUNSs.

**
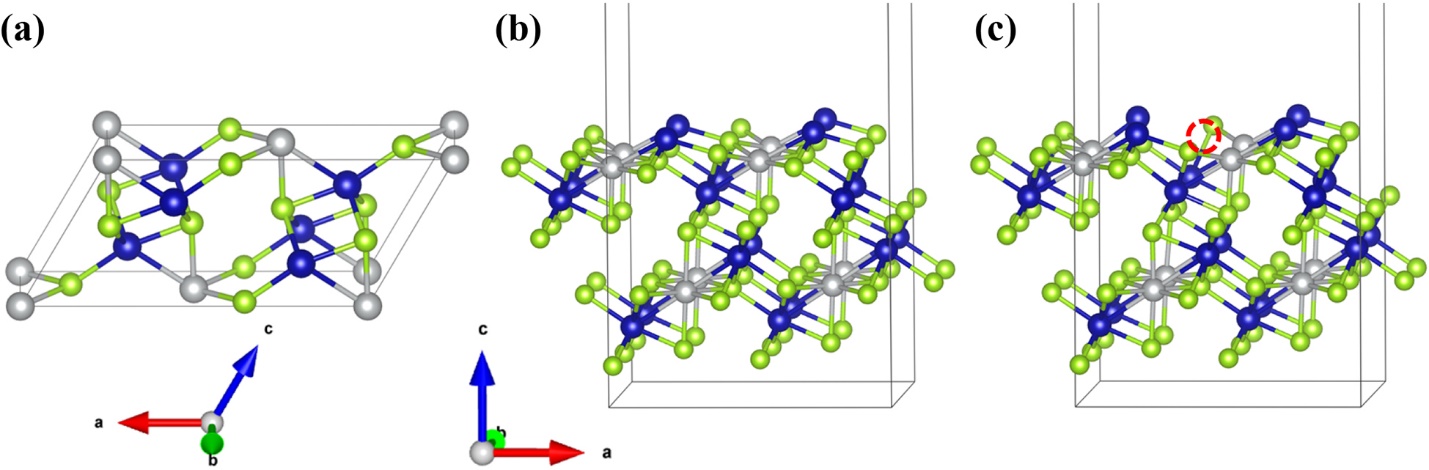
**

**Figure S24.** The geometric structures of (a) NiCo_2_Se_4_ unit cell, (b) slab model for NiCo_2_Se_4_ (001) facet and (c) slab model for V_Se_-NiCo_2_Se_4_ (001) facet.


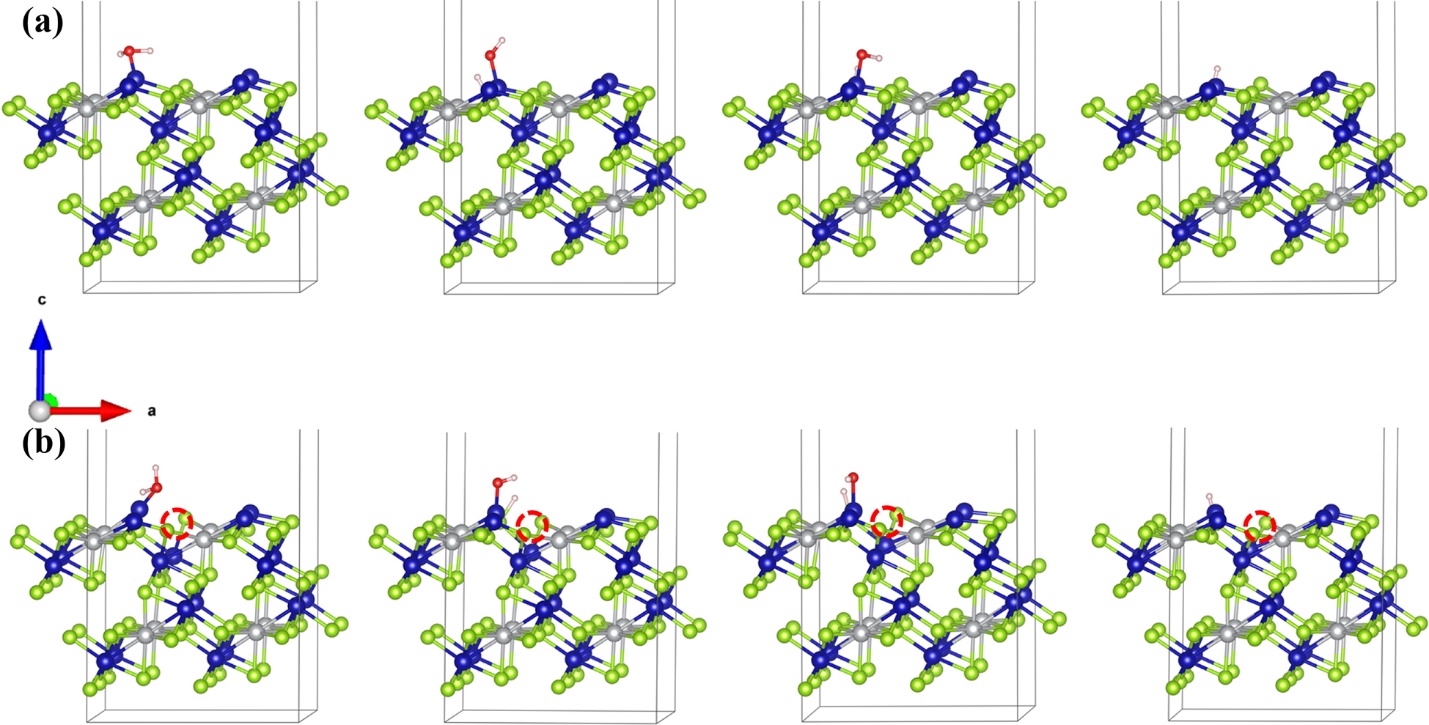


**Figure S25.** The side views of schematic models of HER elementary steps on (a) NiCo_2_Se_4_ (001) and (b) V_Se_-NiCo_2_Se_4_ (001) surfaces.


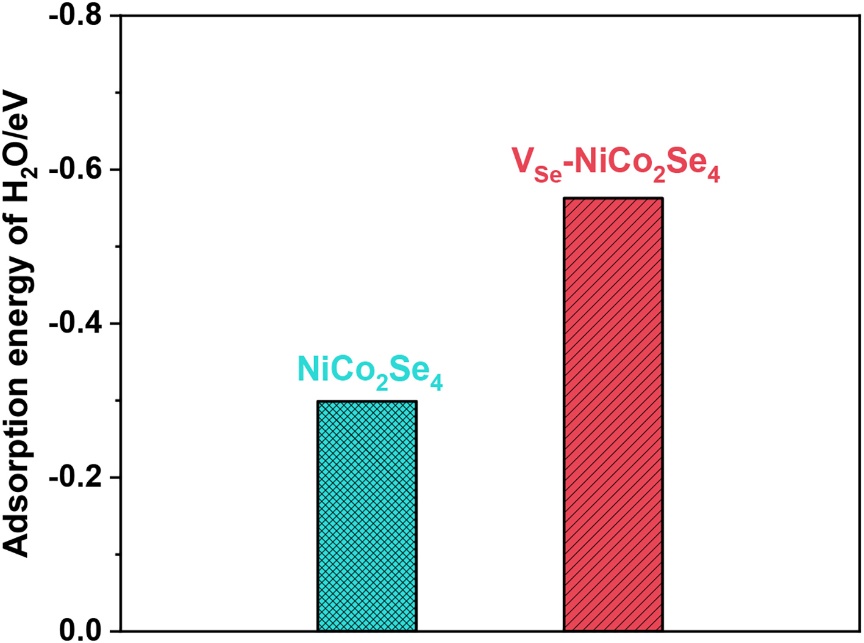


**Figure S26.** The water adsorption energy on NiCo_2_Se_4_ (001) and V_Se_-NiCo_2_Se_4_ (001) surfaces.


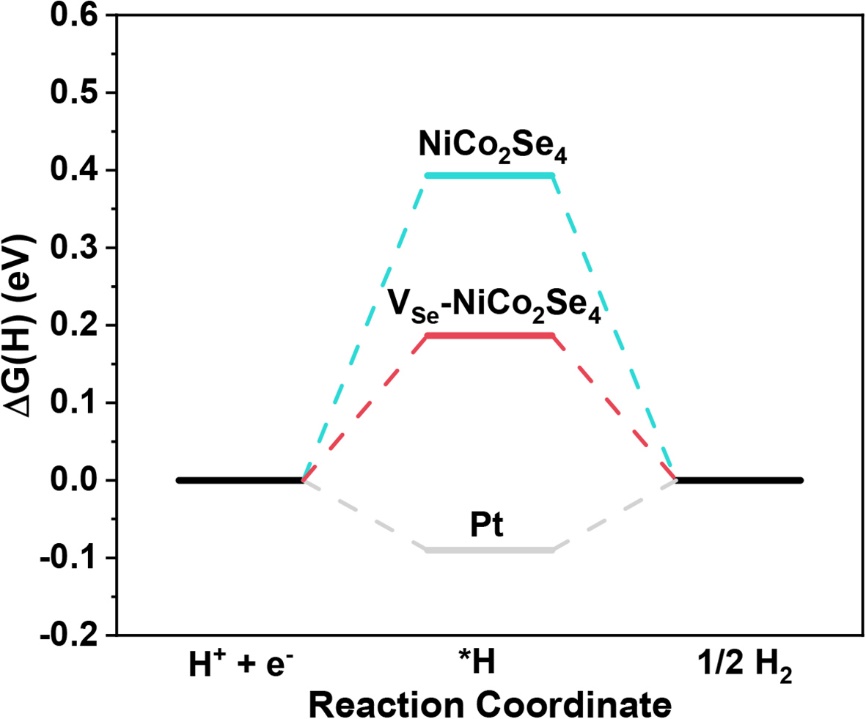


**Figure S27.** Free energy diagram for HER on NiCo_2_Se_4_ (001), V_Se_-NiCo_2_Se_4_ (001) and Pt (111) surfaces.

**
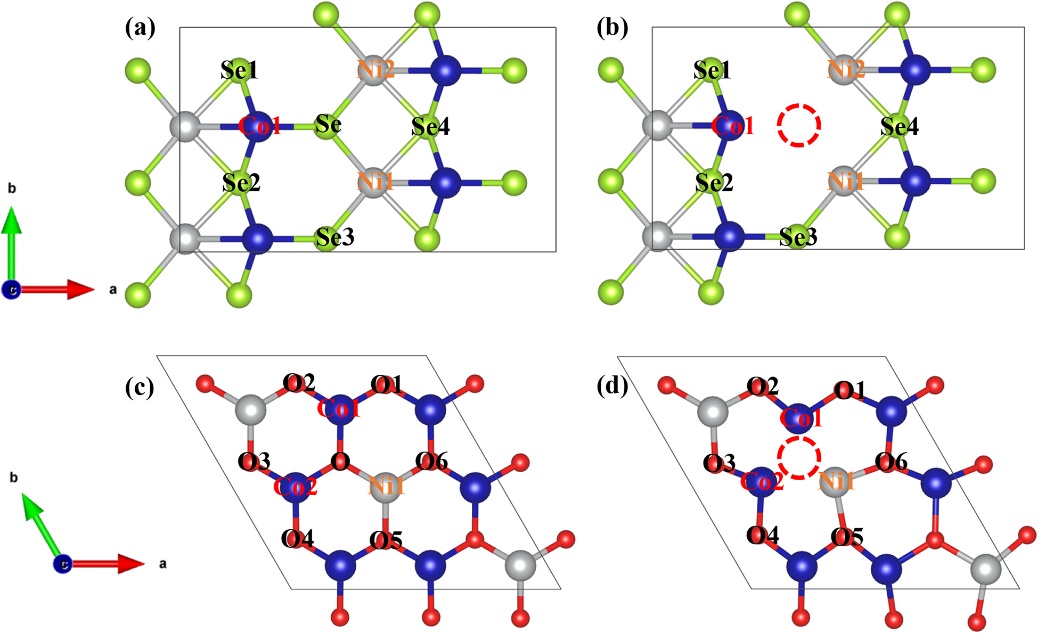
**

**Fig. S28.** The calculate sites for the bader charge analysis in (a) NiCo_2_Se_4_ (001), (b) V_Se_-NiCo_2_Se_4_ (001), (c) NiCoOOH (001) and (d) O_v_-NiCoOOH (001).

**
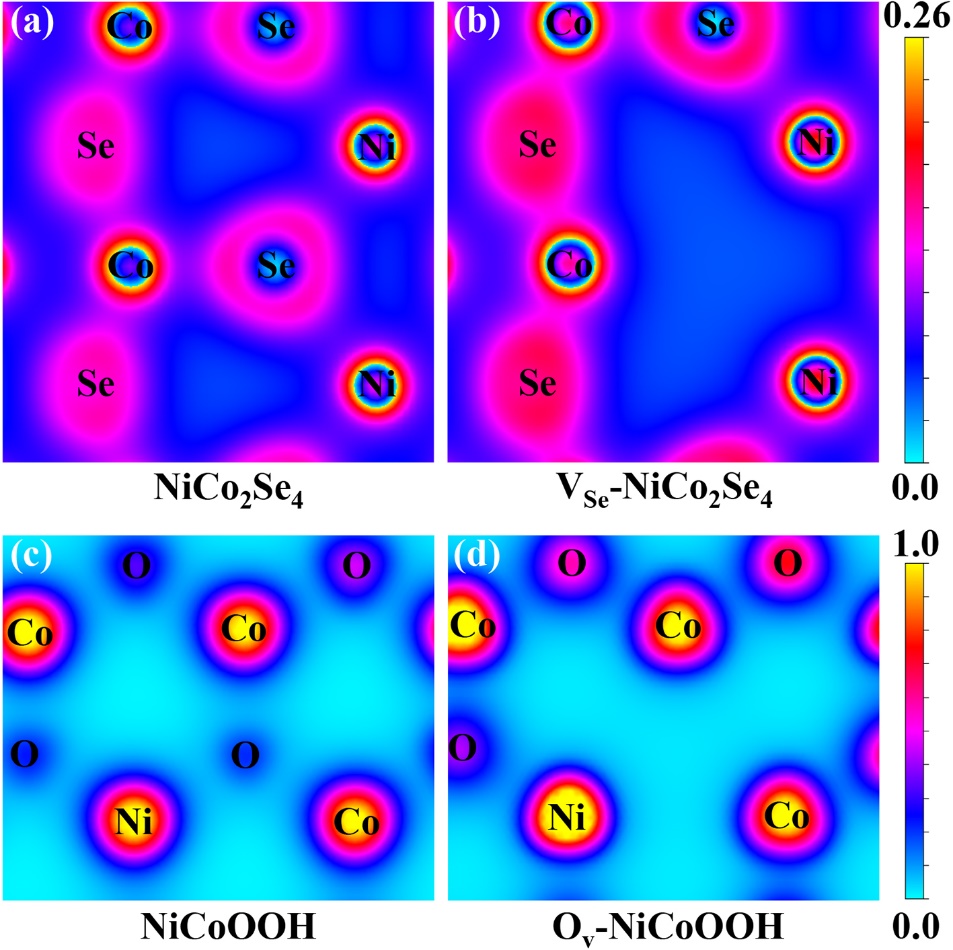
**

**Fig. S29.** Charge density distribution of (a) NiCo_2_Se_4_ (001), (b) V_Se_-NiCo_2_Se_4_ (001), (c) NiCoOOH (001) and (d) O_v_-NiCoOOH (001).


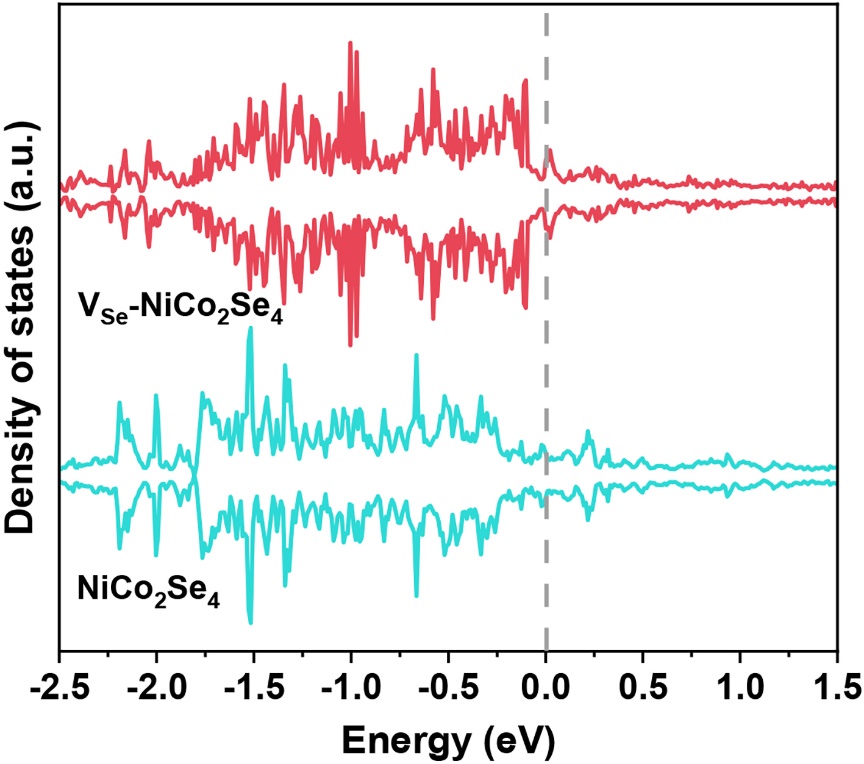


**Figure S30.** Total DOS on NiCo_2_Se_4_ (001) and V_Se_-NiCo_2_Se_4_ (001) surfaces.

**
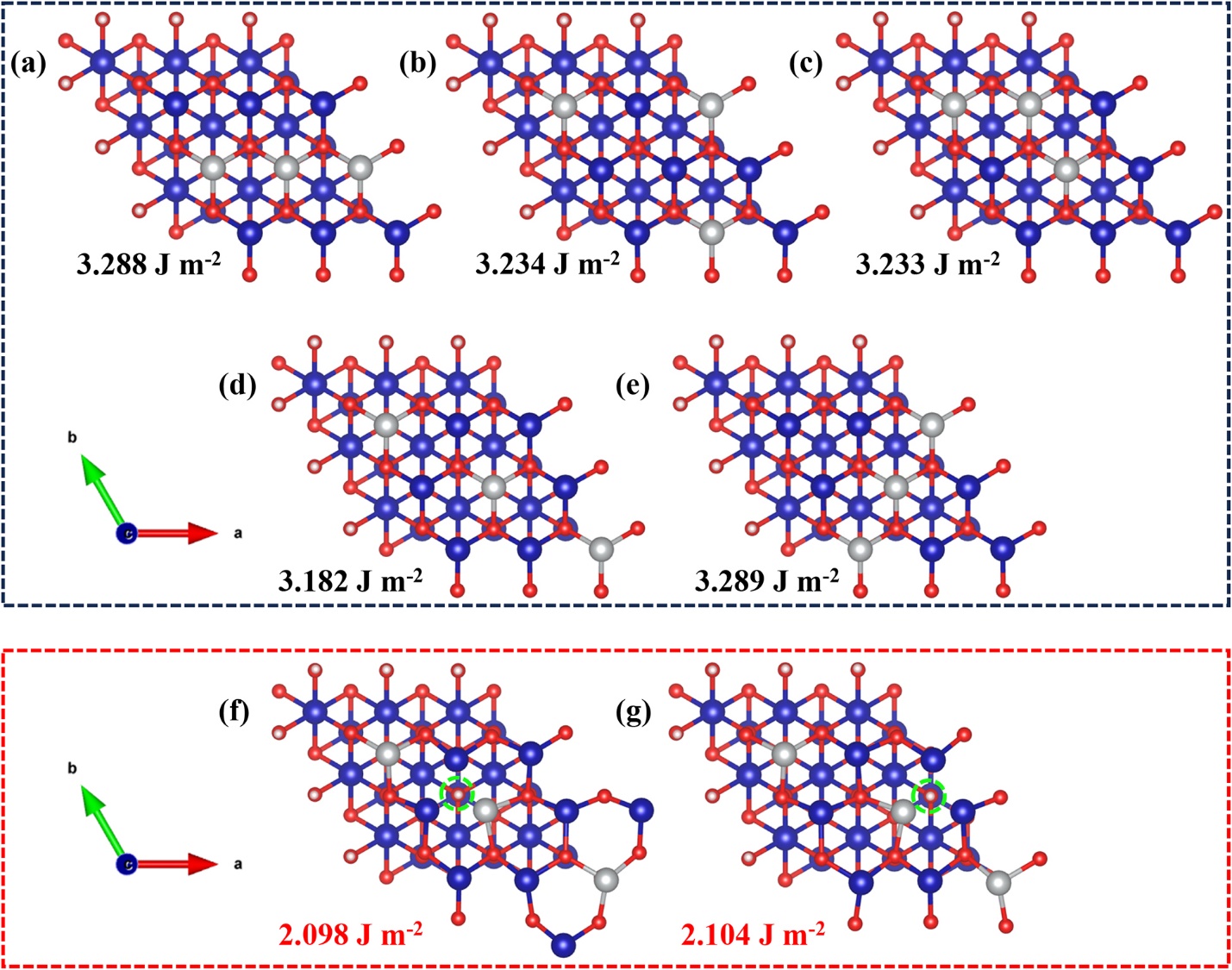
**

**Figure S31.** Optimized atomic structure models (top view) for (a-e) the NiCoOOH (001) and (f, g) O_v_-NiCoOOH (001) surfaces with the different Ni atoms doping positions and different O vacancy positions, respectively. The surface energy is computed to be 3.288, 3.234, 3.233, 3.182 3.289, 2.098 and 2.104 J m^−2^ for (a), (b), (c), (d), (e), (f) and (g), respectively.

**
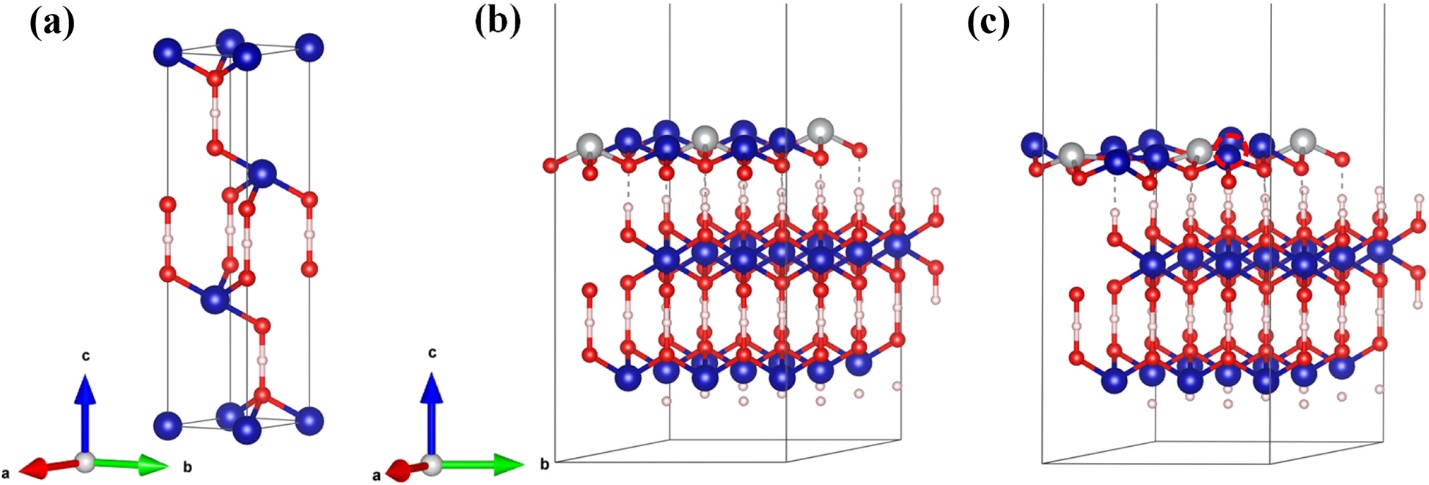
**

**Figure S32.** The geometric structures of (a) CoOOH unit cell, (b) slab model for NiCoOOH (001) facet and (c) slab model for O_v_-NiCoOOH (001) facet.


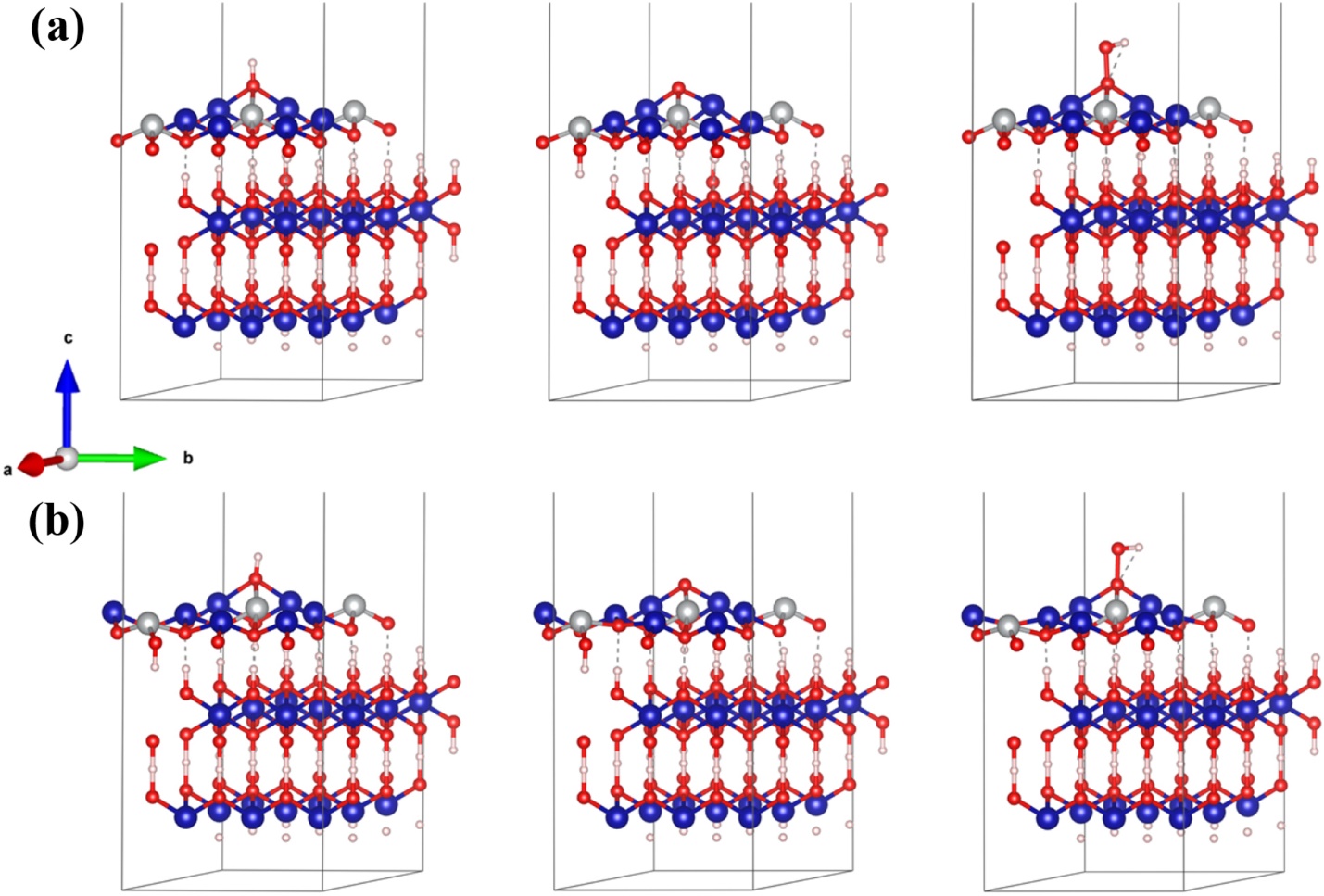


**Figure S33.** Optimized structures after adsorption of *OH, *O and *OOH intermediates on (a) NiCoOOH and (b) O_v_-NiCoOOH. The pink, red, gray, and blue spheres represent H, O, Ni, and Co atoms, respectively.


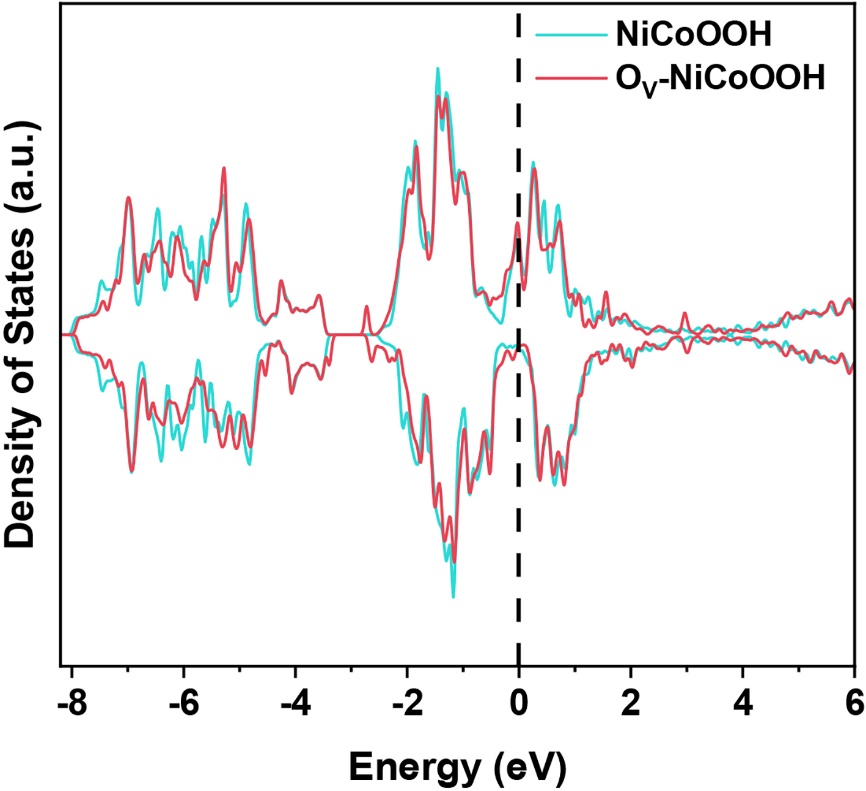


**Figure S34.** Total DOS on NiCoOOH (001) and O_v_-NiCoOOH (001) surfaces.


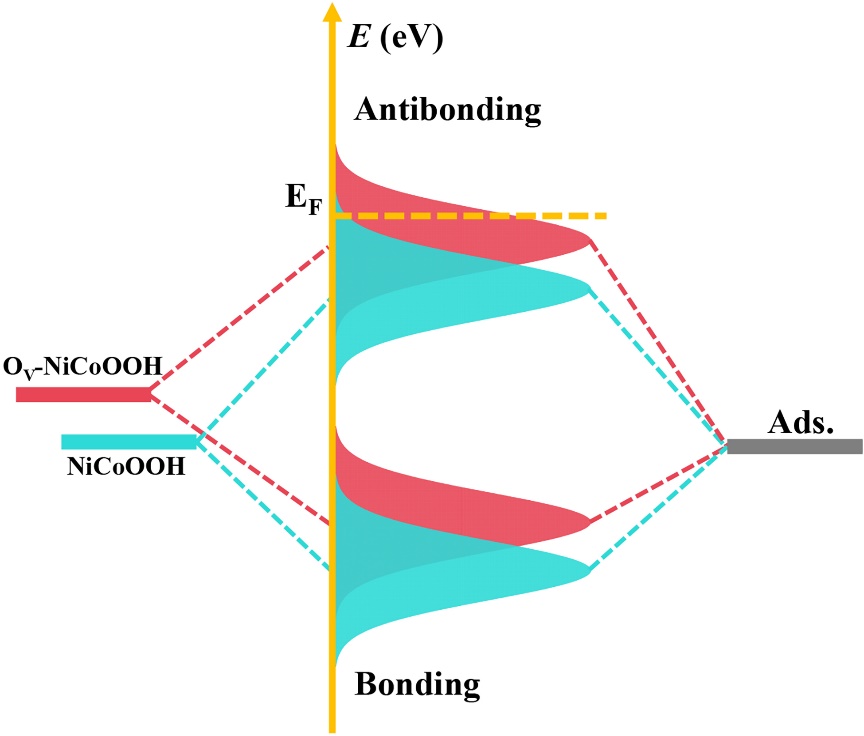


**Figure S35.** The corresponding schematic illustration of bond formation between the reaction surface and the adsorbate (Ads.).


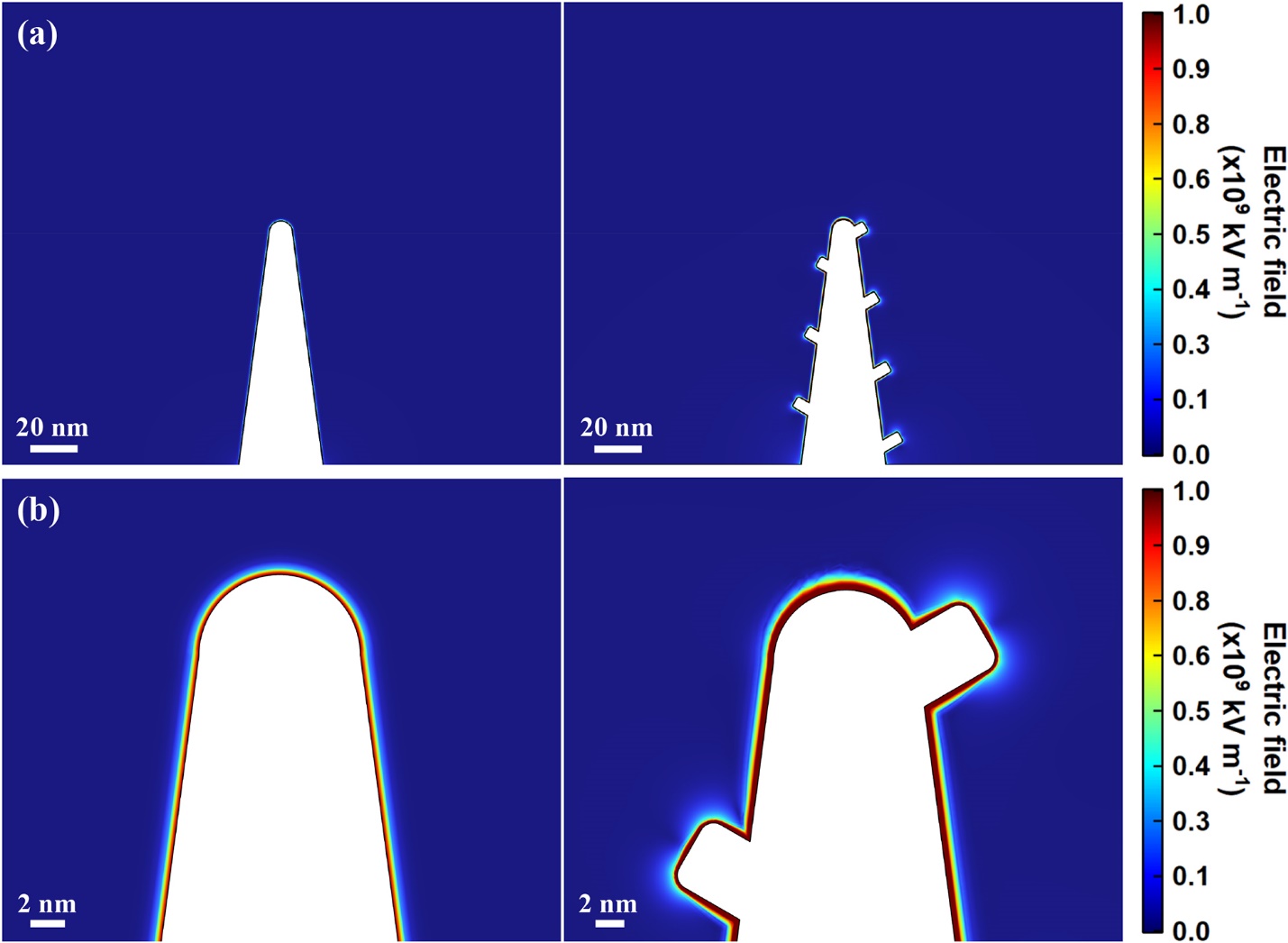


**Figure S36.** (a) The electric field distribution on the surface of nanoneedle (left) and hierarchical ultrathin nanosheet (right). (b) The electric field distribution on the corresponding enlargements of parts (a).


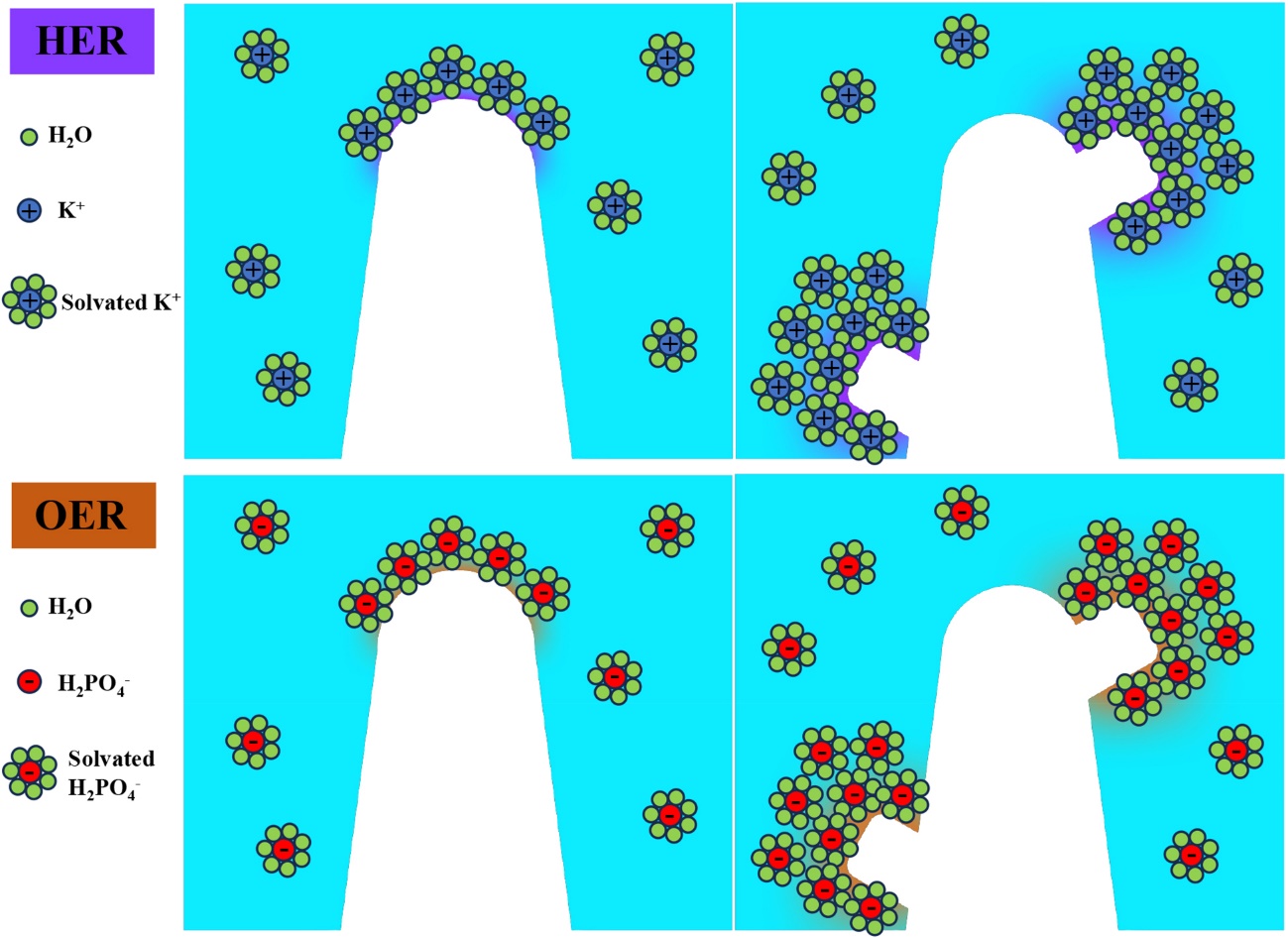


**Figure S37.** The schematic illustration of cation/anion adsorbed on catalysts surface under the effect of electric field.


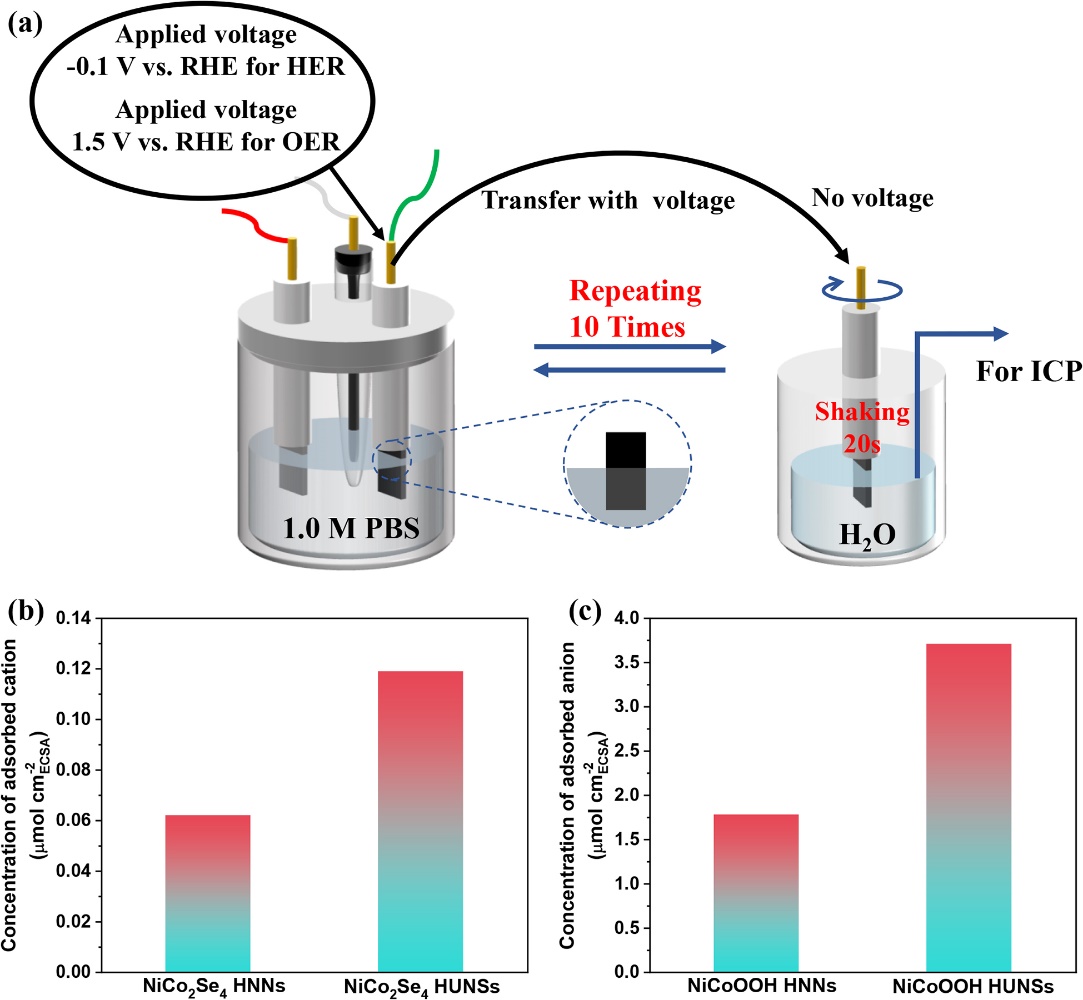


**Figure S38.** (a) Schematic diagram of cation/anion concentration measurement process. ECSA-normalized field-induced concentration of (b) adsorbed cation on NiCo_2_Se_4_ HNNs, NiCo_2_Se_4_ HUNSs and (c) adsorbed anion on NiCoOOH HNNs and NiCoOOH HUNSs.

**Table S1.** Comparison EXAFS fitting parameters at Co and Ni K-edges of NiCo_2_Se_4_ HUNSs and NiCo_2_Se_4_ HNNs.

| **Sample** | **shell** | ***CN^a^*** | ***R*(Å)*^b^*** | ***σ*^2^(Å^2^)*^c^*** | **Δ*E*_0_(eV)*^d^*** | ***R* factor** |
| --- | --- | --- | --- | --- | --- | --- |
| **NiCo_2_Se_4_ HUNSs** | **Co-Se** | 4.8 | 2.38 | 0.0067 | -2.63 | 0.0054 |
| **NiCo_2_Se_4_ HNNs** | **Co-Se** | 5.9 | 2.37 | 0.0061 | -1.04 | 0.0075 |
| **NiCo_2_Se_4_ HUNSs** | **Ni-Se** | 4.9 | 2.39 | 0.0075 | -3.15 | 0.0136 |
| **NiCo_2_Se_4_ HNNs** | **Ni-Se** | 5.9 | 2.38 | 0.0069 | -2.51 | 0.0118 |

*^a^CN*: coordination numbers; *^b^R*: bond distance; *^c^σ*^2^: Debye-Waller factors; *^d^*Δ*E*_0_: the inner potential correction. R factor: goodness of fit.

**Table S2.** Comparison of the electrocatalytic activity of various non-noble metal-based catalysts in the literature with NiCo_2_Se_4_ HUNSs/CFP in this work for the HER in neutral electrolyte.

| Catalysts | η_10_ (mV) | η_100_ (mV) | Tafel slope  (mV dec^−1^) | Ref |
| --- | --- | --- | --- | --- |
| **NiCo_2_Se_4_ HUNSs/CFP** | **36** | **102** | **49** | **In this work** |
| **NiCo_2_Se_4_ HUNSs/NF** | **17** | **92** | — | **In this work** |
| NiCo_2_P_x_/CF | 63 | 173 | 63.3 | *Adv. Mater.,* 2017, **29**, 1605502 |
| FeMoS_4_ NRA/CC | 204 | — | 128 | *Chem. Commun.,* 2017, **53**, 9000--9003 |
| V_0.8_Mo_0.2_Se_2−x_ | 122.3 | — | 66 | *Samll* 2022, **18**, 2204557 |
| S-MoP/CC | 127 | — | 67.1 | *Appl. Catal. B: Environ.,* 2023, **322**, 122131 |
| Ni_0.89_Co_0.11_Se_2_ MNSN/NF | 82 | — | 78 | *Adv. Mater.,* 2017, **29**, 1606521 |
| V−Ni_5_P_4_/NF | 94 | 260 | — | *ACS Appl. Mater. Interfaces* 2020, **12**, 37092−37099 |
| MoN/Co_4_N/CC | 72 | — | 92 | *Chem. Eng. J.*, 2021, **421**, 127757 |
| Co-HNP/CC | 85 | 237 | 38 | *Angew. Chem. Int. Ed.,* 2016, **55**, 6725–6729 |
| Ni_0.1_Co_0.9_P/CFP | 125 | — | 103 | *Angew. Chem. Int. Ed.,* 2018, **57**, 15445 –15449 |
| np-Co_9_S_4_P_4_ | 87 | 174 | 51 | *ACS Appl. Mater. Interfaces* 2019, **11**, 3880−3888 |
| Fe-Mo_2_C@NCF | 130 | 275 | 109 | *J. Mater. Chem. A,* 2020, **8**, 19879–19886 |
| N, Mn-MoS_2_/NF | 70 | — | 65 | *ACS Catal.,* 2018, **8**, 7585−7592 |
| CeO_2_/Co_4_N/CC | 75 | — | 112 | *Appl. Catal B: Environ.,* 2020, **277**, 119282 |
| P-MoP/Mo_2_N | 91 | 290 | 51 | *Angew. Chem. Int. Ed.,* 2021, **133**, 6747–6755 |
| Cu_2−x_Se@(Co,Cu)Se_2_ | 106 | — | 81 | *Nanoscale* 2021, **13**, 1134–1143 |
| P_3_-MNS_3_/NF | 128 | 242 | 112 | *ACS Sustainable Chem. Eng.* 2021, **9**, 10601−10610 |
| 3LH-Co/CoP@NC/CC | 145 | — | 129 | *Sci. China Chem.,* 2022, **65**, 619–629 |
| *p*Fe/FeP | 125 | 380 | 66 | *Chem. Eng. J.,* 2021, **408**, 127330 |
| WS_2_/Co_9_S_8_/Co_4_S_3_ | 208 | — | 125.8 | *ACS Appl. Mater. Interfaces* 2023,**15**,11765−1177 |
| NiCo_2_Te_4_/PTCDA | 60 | 120 | 38 | *Appl. Catal. B: Environ.,* 2019, **254**, 424–431 |
| MoP NA/CC | 187 | 379 | 94 | *Appl. Catal. B: Environ.,* 2016, **196**, 193–198 |
| v-NiFe LDH/NF | 87 | — | 46.3 | *ACS Energy Lett.,* 2019, **4**, 1412−1418 |
| CoO/CoSe_2_/Ti | 337 | — | 131 | *Adv. Sci.,* 2016, **3**, 1500426 |
| CoP/CeO_2_−FeO_x_H/NF | 149 | 300 | 101 | *ACS Sustainable Chem. Eng.,* 2021, **9**, 11981−11990 |
| Co-C-N | 273 | — | 107 | *J. Am. Chem. Soc.,* 2015, **137**, 15070−15073 |
| NiMo@C/NF | 98 | — | 71.3 | *ACS Appl. Mater. Interfaces* 2023,**15**,20130−2014 |
| Ni_0.33_Co_0.67_S_2_/Ti | 72 | — | 67.8 | *Adv. Energy Mater.,* 2015, **5**, 1402031 |
| CuO_x_@NiMnO_x_/CF | 80.7 | 556 | 77.6 | *J. Mater. Chem. A,* 2020, **8**, 16463–16476 |
| CoP/CC | 106 | — | 93 | *J. Am. Chem. Soc.,* 2014, **136**, 7587−7590 |
| WC-W_2_C/HCDs | 148 | — | 112.9 | *Chem. Eng. J.*, 2023, **462**, 142132 |
| Ni_3_S_2_/Ni | 220 | 396 | 118 | *Int J Hydrogen Energy* 2015, **240**, 4727−4732 |
| FePSe_3_/NC | 140.1 | — | 167 | *Nano Energy* 2019, **57**, 222–229 |
| Mn-Co-P/Ti | 86 | — | 82 | *ACS Catal.,* 2017, **7**, 98−102 |
| CoP/Ni_2_P/MoS_2_–CC | — | 335 | 63 | *ACS Appl. Energy Mater.,* 2023, **6**, 9577−9584 |
| Am-Mo-NiS_0.5_Se_0.5_/NF | 48 | — | 52 | *Angew. Chem. Int. Ed.,* 2023, **62**, e202215256 |
| MoS_2_/NLG-3/CFP | 142 | — | 72.9 | *ACS Catal.,* 2021, **11**, 4486−4497 |
| CoBDC/MXene | 76 | — | 71 | *Adv. Funct. Mater.,* 2023, **33**, 2210322 |
| N@Ni_3_N–Ni-6/CC | 85 | 295 | 97 | *J. Mater. Chem. A,* 2019, **7**, 15823–15830 |

**Table S3.** Comparison of the electrocatalytic activity of various non-noble metal-based catalysts in the literature with NiCoOOH HUNSs/CFP in this work for the OER in neutral electrolyte.

| Catalysts | η_10_ (mV) | η_100_ (mV) | Tafel slope  (mV dec^−1^) | Ref |
| --- | --- | --- | --- | --- |
| **NiCoOOH HUNSs/CFP** | — | **269** | **68** | **In this work** |
| **NiCoOOH HUNSs/NF** | **—** | **214** | — | **In this work** |
| Ni_3_Se_4_/Ni | 480 | — | 116 | *ACS Appl. Mater. Interfaces* 2017, **9**, 8714−8728 |
| Cu_2−x_Se@(Co,Cu)Se_2_ | 396 | — | 102 | *Nanoscale* 2021, **13**, 1134–1143 |
| N-Fe_2_PO_5–x_-OT/NF | 315 | 470 | 94 | *Adv. Funct. Mater.,* 2018, **28**, 1801397 |
| Ni-CoOOH/NF | 410 | — | 187 | *Chem. Eng. J.*, 2020, **398**, 125537 |
| Cu_0.08_Co_0.92_P/CP | 411 | — | 101.4 | *Appl. Catal. B: Environ.,* 2020, **265**, 118555 |
| Sm−LaCoO_3_ | 530 | — | 135.5 | *J. Am. Chem. Soc.,* 2022, **144**, 13163−13173 |
| Ti@Co_0.85_Se | 500 | — | 153 | *Nano Energy* 2017, **39**, 321–327 |
| Co(OH)_2_/CFP | 396 | 507 | 112 | *Angew. Chem. Int. Ed.,* 2023, **62**, e202308335 |
| Co-(NiFe)N@NiS_x_@NF | — | 425 | 68.2 | *Appl. Catal. B: Environ.,* 2023, **330**, 122599 |
| Co-Mo_2_C@NC | 440 | — | 156 | *J. Colloid Interface Sci.,* 2018, **532**, 774–781 |
| CoSAs-MoS_2_/TiN/CC | 508 | — | 183.1 | *Adv. Funct. Mater.,* 2021, **31**, 2100233 |
| Zn_0.075_, S–Co_0.925_P/CP | 391 | — | 99.7 | *J. Mater. Chem. A,* 2019, **7**, 22453–22462 |
| Ni(S_0.5_Se_0.5_)_2_ | 501 | — | 94 | *J. Mater. Chem. A,* 2019, **7**, 16793–16802 |
| Co_3_O_4_@Fe-B-O/NF | 384 | — | 112 | *J. Colloid Interface Sci.,* 2023, **646**, 452–460 |
| Co-Pi NA/Ti | 380 | — | / | *Angew. Chem. Int. Ed.,* 2017, **56**, 1064 –1068 |
| CuO@Co_2_P/CF | 260 | 472 | 98.3 | *Colloid Surface A.,* 2023, **673**, 131802 |
| CoP NA/CC | 536 | — | 257 | *ChemElectroChem* 2017, **4**, 1840–1845 |
| S-NiFe_2_O_4_/NF | 494 | — | 118.1 | *Nano Energy* 2017, **40**, 264–273 |
| CoFe-LDH_0.5_ fiber | 300 | — | 84.14 | *ACS Appl. Energy Mater.,* 2022, **5**, 11483−11497 |
| Co_2_Al_1_ LDH/CC | 200 | — | 256 | *ACS Appl. Mater. Interfaces* 2023, **15**, 11621−11630 |
| CoO/CoSe_2_/Ti | 510 | — | 137 | *Adv. Sci.,* 2016, **3**, 1500426 |
| Co@CoO-PNC/CC | 371 | — | 211 | *J. Alloys Compd.,* 2021, **877**, 160279 |
| NiFeCu/NF | 385 | — | 164 | *ACS Catal.,* 2020, **10**, 9725−9734 |
| (Fe_0.5_Ni_0.5_)_2_P/NF | 396 | — | 182 | *Nano Energy* 2017, **38**, 553–560 |
| CoP@CoOOH/CP | 318 | — | 127.4 | *Small* 2022, **18**, 2106012 |
| CuO_x_@NiMnO_x_/CF | 390 | — | 101.6 | *J. Mater. Chem. A,* 2020, **8**, 16463–16476 |
| Ni–Fe–Mg Oxyhydroxide | 514 | — | 150 | *Adv. Mater.,* 2020, **32**, 1906806 |
| 3.47% Co/ZnO/CFP | 450 | — | 106 | *ACS Sustainable Chem. Eng.,* 2019, **7**, 18055−18060 |
| Am-Mo-NiS_0.5_Se_0.5_/NF | 238 | — | 48 | *Angew. Chem. Int. Ed.,* 2023, **62**, e202215256 |
| Ni(OH)_2_/Ni:Pi/NF | 340 | — | 175 | *Inorg. Chem. Front.,* 2019, **6**, 3093–3096 |

**Table S4.** Comparison EXAFS fitting parameters at Co and Ni K-edges of NiCoOOH HUNSs and NiCoOOH HNNs.

| **Sample** | **shell** | ***CN^a^*** | ***R*(Å)*^b^*** | ***σ*^2^(Å^2^)*^c^*** | **Δ*E*_0_(eV)*^d^*** | ***R* factor** |
| --- | --- | --- | --- | --- | --- | --- |
| **NiCoOOH HUNSs** | **Co-O** | 4.6 | 1.90 | 0.0042 | -4.72 | 0.0094 |
|  | **Co-Co** | 3.1 | 2.85 | 0.0068 |  |  |
| **NiCoOOH HNNs** | **Co-O** | 5.1 | 1.90 | 0.0043 | -4.86 | 0.0079 |
|  | **Co-Co** | 3.9 | 2.85 | 0.0072 |  |  |
| **NiCoOOH HUNSs** | **Ni-O** | 4.7 | 2.01 | 0.0039 | -4.22 | 0.0086 |
| **NiCoOOH HNNs** | **Ni-O** | 5.3 | 2.01 | 0.0042 | -4.40 | 0.0099 |

*^a^CN*: coordination numbers; *^b^R*: bond distance; *^c^σ*^2^: Debye-Waller factors; *^d^*Δ*E*_0_: the inner potential correction. R factor: goodness of fit.

**Table S5.** Bader charge analysis of V_Se_-NiCo_2_Se_4_ (001), NiCo_2_Se_4_ (001), Ov-NiCoOOH (001) and NiCoOOH (001).

| **Samples** | **Ni1** | | **Ni2** | | **Co1** | | **Se1** | | **Se2** | | | **Se3** | | **Se4** | |
| --- | --- | --- | --- | --- | --- | --- | --- | --- | --- | --- | --- | --- | --- | --- | --- |
| **V_Se_-NiCo_2_Se_4_ (001)** | -0.081 | | -0.079 | | -0.132 | | 0.215 | | 0.180 | | | 0.192 | | 0.167 | |
| **NiCo_2_Se_4_ (001)** | -0.126 | | -0.126 | | -0.163 | | 0.184 | | 0.167 | | | 0.164 | | 0.163 | |
| **Samples** | **Ni1** | **Co1** | | **Co2** | | **O1** | | **O2** | | **O3** | **O4** | | **O5** | | **O6** |
| **O_v_-NiCoOOH (001)** | -0.019 | -0.158 | | -0.163 | | 0.662 | | 0.738 | | 0.791 | 0.795 | | 0.800 | | 0.672 |
| **NiCoOOH (001)** | -0.094 | -0.306 | | -0.620 | | 0.602 | | 0.350 | | 0.637 | 0.394 | | 0.599 | | 0.344 |
